# Fatty acid oxidation-induced HIF-1α activation facilitates hepatic urate synthesis through upregulating NT5C2 and XDH

**DOI:** 10.1093/lifemeta/loae018

**Published:** 2024-05-17

**Authors:** Ningning Liang, Xuan Yuan, Lili Zhang, Xia Shen, Shanshan Zhong, Luxiao Li, Rui Li, Xiaodong Xu, Xin Chen, Chunzhao Yin, Shuyuan Guo, Jing Ge, Mingjiang Zhu, Yongzhen Tao, Shiting Chen, Yongbing Qian, Nicola Dalbeth, Tony R Merriman, Robert Terkeltaub, Changgui Li, Qiang Xia, Huiyong Yin

**Affiliations:** CAS Key Laboratory of Nutrition, Metabolism, and Food Safety, Shanghai Institute of Nutrition and Health, Chinese Academy of Sciences (CAS), Shanghai 200031, China; University of Chinese Academy of Sciences, Beijing 100049, China; Department of Biomedical Sciences, Jockey Club College of Veterinary Medicine and Medicine, Tung Biomedical Science Center, State Key Laboratory of Marine Pollution (SKLMP), The Shenzhen Research Institute and Futian Research Institute, City University of Hong Kong, Hong Kong 999077, China; Institute of Metabolic Diseases, Qingdao University, Qingdao, Shandong 266003, China; Shandong Provincial Key Laboratory of Metabolic Diseases, Qingdao Key Laboratory of Gout, Affiliated Hospital of Qingdao University Medical College, Qingdao University, Qingdao, Shandong 266071, China; CAS Key Laboratory of Nutrition, Metabolism, and Food Safety, Shanghai Institute of Nutrition and Health, Chinese Academy of Sciences (CAS), Shanghai 200031, China; University of Chinese Academy of Sciences, Beijing 100049, China; CAS Key Laboratory of Nutrition, Metabolism, and Food Safety, Shanghai Institute of Nutrition and Health, Chinese Academy of Sciences (CAS), Shanghai 200031, China; University of Chinese Academy of Sciences, Beijing 100049, China; School of Life Science and Technology, ShanghaiTech University, Shanghai 201210, China; CAS Key Laboratory of Nutrition, Metabolism, and Food Safety, Shanghai Institute of Nutrition and Health, Chinese Academy of Sciences (CAS), Shanghai 200031, China; University of Chinese Academy of Sciences, Beijing 100049, China; Department of Biomedical Sciences, Jockey Club College of Veterinary Medicine and Medicine, Tung Biomedical Science Center, State Key Laboratory of Marine Pollution (SKLMP), The Shenzhen Research Institute and Futian Research Institute, City University of Hong Kong, Hong Kong 999077, China; CAS Key Laboratory of Nutrition, Metabolism, and Food Safety, Shanghai Institute of Nutrition and Health, Chinese Academy of Sciences (CAS), Shanghai 200031, China; University of Chinese Academy of Sciences, Beijing 100049, China; CAS Key Laboratory of Nutrition, Metabolism, and Food Safety, Shanghai Institute of Nutrition and Health, Chinese Academy of Sciences (CAS), Shanghai 200031, China; University of Chinese Academy of Sciences, Beijing 100049, China; School of Life Science and Technology, ShanghaiTech University, Shanghai 201210, China; CAS Key Laboratory of Nutrition, Metabolism, and Food Safety, Shanghai Institute of Nutrition and Health, Chinese Academy of Sciences (CAS), Shanghai 200031, China; University of Chinese Academy of Sciences, Beijing 100049, China; CAS Key Laboratory of Nutrition, Metabolism, and Food Safety, Shanghai Institute of Nutrition and Health, Chinese Academy of Sciences (CAS), Shanghai 200031, China; University of Chinese Academy of Sciences, Beijing 100049, China; CAS Key Laboratory of Nutrition, Metabolism, and Food Safety, Shanghai Institute of Nutrition and Health, Chinese Academy of Sciences (CAS), Shanghai 200031, China; University of Chinese Academy of Sciences, Beijing 100049, China; School of Life Science and Technology, ShanghaiTech University, Shanghai 201210, China; CAS Key Laboratory of Nutrition, Metabolism, and Food Safety, Shanghai Institute of Nutrition and Health, Chinese Academy of Sciences (CAS), Shanghai 200031, China; University of Chinese Academy of Sciences, Beijing 100049, China; School of Life Science and Technology, ShanghaiTech University, Shanghai 201210, China; CAS Key Laboratory of Nutrition, Metabolism, and Food Safety, Shanghai Institute of Nutrition and Health, Chinese Academy of Sciences (CAS), Shanghai 200031, China; University of Chinese Academy of Sciences, Beijing 100049, China; CAS Key Laboratory of Nutrition, Metabolism, and Food Safety, Shanghai Institute of Nutrition and Health, Chinese Academy of Sciences (CAS), Shanghai 200031, China; University of Chinese Academy of Sciences, Beijing 100049, China; CAS Key Laboratory of Nutrition, Metabolism, and Food Safety, Shanghai Institute of Nutrition and Health, Chinese Academy of Sciences (CAS), Shanghai 200031, China; University of Chinese Academy of Sciences, Beijing 100049, China; CAS Key Laboratory of Nutrition, Metabolism, and Food Safety, Shanghai Institute of Nutrition and Health, Chinese Academy of Sciences (CAS), Shanghai 200031, China; University of Chinese Academy of Sciences, Beijing 100049, China; Department of Liver Surgery, Renji Hospital, Shanghai Jiao Tong University School of Medicine, Shanghai 200127, China; Department of Medicine, Faculty of Medical and Health Sciences, University of Auckland, Auckland 1142, New Zealand; Department of Biochemistry, University of Otago, Dunedin 9016, New Zealand; Division of Clinical Immunology and Rheumatology, University of Alabama at Birmingham, Birmingham, Alabama 35294, United States; VA San Diego Healthcare System, San Diego, La Jolla, CA 92037, United States; School of Medicine, University of California San Diego, La Jolla, CA 92037, United States; Institute of Metabolic Diseases, Qingdao University, Qingdao, Shandong 266003, China; Shandong Provincial Key Laboratory of Metabolic Diseases, Qingdao Key Laboratory of Gout, Affiliated Hospital of Qingdao University Medical College, Qingdao University, Qingdao, Shandong 266071, China; Department of Liver Surgery, Renji Hospital, Shanghai Jiao Tong University School of Medicine, Shanghai 200127, China; CAS Key Laboratory of Nutrition, Metabolism, and Food Safety, Shanghai Institute of Nutrition and Health, Chinese Academy of Sciences (CAS), Shanghai 200031, China; University of Chinese Academy of Sciences, Beijing 100049, China; Department of Biomedical Sciences, Jockey Club College of Veterinary Medicine and Medicine, Tung Biomedical Science Center, State Key Laboratory of Marine Pollution (SKLMP), The Shenzhen Research Institute and Futian Research Institute, City University of Hong Kong, Hong Kong 999077, China; School of Life Science and Technology, ShanghaiTech University, Shanghai 201210, China

**Keywords:** dyslipidemia, hyperuricemia, purine, hypoxanthine, metabolic flux

## Abstract

Dyslipidemia affects approximately half of all people with gout, and prior Mendelian randomization analysis suggested a causal role for elevated triglycerides in hyperuricemia (HU), but the underlying mechanisms remain elusive. We hypothesize that dyslipidemia promotes hepatic urate biosynthesis in HU and gout and fatty acid (FA) oxidation (FAO) drives this process. Here we developed a targeted metabolomics to quantify major metabolites in purine metabolic pathway in the sera of a human cohort with HU, gout, and normaluricemic controls. We found that the levels of major purine metabolites and multiple FAs were significantly elevated in HU and gout groups compared to normouricemic controls, whereas hypoxathine showed opposite trend. Furthermore, the levels of multiple serum FAs were positively correlated with urate, xanthine, and inosine but negatively with hypoxanthine, which was also observed in a murine model of high-fat diet-induced HU. Using a stable isotope-labeled metabolic flux assay, we discovered that exogenous hypoxanthine plays a key role in urate synthesis. Moreover, FAO-induced hypoxia-inducible factor 1 alpha (HIF-1α) activation upregulated 5ʹ-nucleotidase II (NT5C2) and xanthine dehydrogenase (XDH) levels to facilitate hypoxanthine uptake from the blood to the liver and activation of urate biosynthesis. Our findings were further supported by data in human hepatocytes and 50 paired serum and liver tissues from liver transplant donors. Together, this study uncovers a mechanism by which FAO promotes hepatic urate synthesis by activating HIF-1α-NT5C2/XDH pathways, directly linking lipid metabolism to HU.

## Introduction

Urate is the end product of human purine metabolism. Dysregulation of purine metabolism may lead to elevated serum urate levels greater than the saturated urate concentration of 420 μmol/L, termed hyperuricemia (HU) [1]. The prevalence of HU in different ethnic groups ranges from 2.6% to 36%, and is much more common in men than in women, with the ratio of male to female ranges between 2:1 and 5:1 [2–5]. In China, the prevalence of HU is around 13.3%, which accounts for more than 160 million adults [6]. The predominant cause of HU is urate underexcretion by the kidney and/or the gut, but many patients have both urate overproduction and underexcretion, and a smaller fraction has urate overproduction in isolation [7].

Urate overproduction primarily occurs in the liver [7]. In this process, urate is generated from the metabolism of purines by *de novo* purine biosynthesis (DNPB) and purine nucleotide salvage [8]. Previous studies indicated that hepatocytes generate urate predominantly through the salvage pathway in the presence of purines, whereas DNPB was only present in an appreciable amount in purine-poor media [9]. Xanthine oxidoreductase (XOR), the key enzyme catalyzing the oxidation of hypoxanthine and xanthine to urate, is the drug target for the most widely used urate-lowering therapy (ULT) medications for gout and HU, allopurinol and to a lesser extent febuxostat [10]. XOR exists in two inter-convertable forms in mammals: xanthine oxidase (XO) and xanthine dehydrogenase (XDH), while in the normal liver, it mainly exists in the XDH form [11].

HU is a component of metabolic syndrome, and each of the components of metabolic syndrome, including dyslipidemia, commonly affects gout patients [12, 13]. In this context, dyslipidemia affects approximately half of all gout patients [14]. Furthermore, prior Mendelian randomization analysis identified a potential causal role for elevated triacylglycerols (triglycerides or TAGs) in HU [15], and was supported by data from several human cohorts [16, 17]. The molecular mechanism linking HU and hyperlipidemia may be partially genetic [15], but, on the whole, remains unclear. Early studies found that lowering serum TAG levels significantly reduces serum urate levels in patients with hypertriglyceridemia [18]. Recent studies found that non-alcoholic fatty liver disease (NAFLD) significantly increases the risk of HU. Interestingly, the early stage of NAFLD, hepatosteatosis, is often accompanied by enhanced fatty acid (FA) oxidation (FAO) with HU [19, 20], while XOR can also promote activation of the nucleotide-binding oligomerization domain (NOD)-like receptor family pyrin domain containing 3 (NLRP3) inflammasome, which predominantly mediates non-alcoholic hepatotitis (NASH) [21, 22]. However, how XOR is activated in NAFLD and other human conditions associated with dyslipidemia remains to be explored.

This study tested the hypothesis that dyslipidemia could promote hepatic urate biosynthesis in people with HU and gout. By doing so, we investigated whether FAO in the context of dyslipidemia directly led to hepatic urate production and HU in humans. We further studied the correlations of FA levels, as a marker for TAG levels, and major metabolites in the purine salvage pathway in a cohort comprised of people with HU, gout, and normouricemia. The underlying mechanisms of FAO and hepatic urate biosynthesis were examined by both cell and animal models, combined with stable isotope-labeled metabolic flux assays.

## Results

### Unique pattern of hypoxanthine compared to other major purine metabolites identified by targeted serum metabolomics

**Figure 1:**
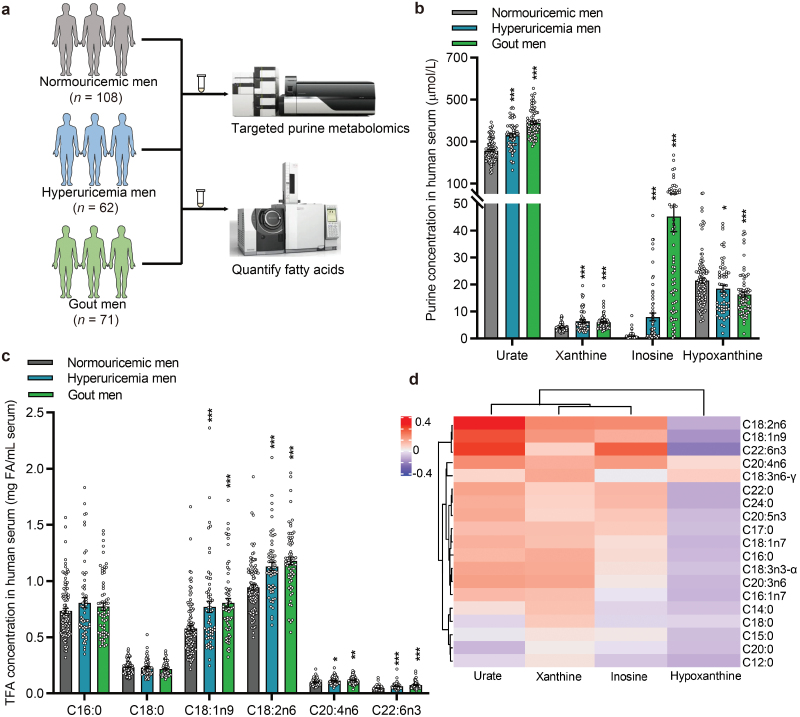
The levels of FAs are positively correlated with urate levels while negatively correlated with hypoxanthine levels in human serum identified by targeted metabolomic analysis of purine metabolites and FAs. (a) Flow chart shows the samples used for targeted purine metabolomics and FA detection. (b and c) Quantitative results of major metabolites related to purine (b) and FAs (c) in human serum (*n *= 241). (d) Heatmap analysis of purine metabolites relative to FAs in human serum (*n* = 241). TFA, total fatty acid. ^*^P < 0.05, ^**^P < 0.01, ^***^P < 0.001 by unpaired Mann–Whitney test (b and c).

To investigate the interplay between the purine metabolic pathways and lipid metabolism, we developed a targeted metabolomic method to quantify the 13 major purine metabolites in the purine salvage and urate production pathways using liquid chromatography-mass spectrometry (LC-MS) (Supplementary Fig. S1), and total fatty acids (TFAs), markers of the TAG levels, were analyzed by gas chromatography-mass spectrometry (GC-MS). These techniques were applied to the serum samples of a cohort consisting of 241 male subjects (108 with normouricemia, 62 with HU, and 71 with gout) (Fig. 1a). Recently, we profiled the differential metabolites and metabolic pathways between HU and gout, using non-targeted metabolomics of sera from this cohort [23]. Clinical characteristics of the participants are shown in Table 1. Compared with the normouricemic group, the levels of most abundant purine metabolites, including urate, xanthine, and inosine in the HU and gout groups were all significantly higher, whereas the levels of hypoxanthine were significantly decreased in the HU and gout groups (Fig. 1b). The levels of major FAs, such as C18:1n9, C18:2n6, C20:4n6, and C22:6n3, were significantly higher in the HU and gout subjects compared with the normouricemia ones (Fig. 1c; Supplementary Fig. S2a and b). In addition, the levels of most FAs were positively correlated with urate, xanthine, and inosine, but negatively correlated with hypoxanthine (Fig. 1d). The collective human data suggested that purine and FA metabolism are closely linked. Moreover, the unique pattern of hypoxanthine compared to other major purine metabolites prompted us to hypothesize that higher levels of FAs could facilitate the conversion of hypoxanthine to urate, and consequently decrease the levels of hypoxanthine in the serum, accompanied by increased levels of other major purine metabolites.

**Table 1.** Clinical and demographic characteristics of human participants.

	Normouricemic men (*n* = 108)	Hyperuricemic men (*n* = 62)	Gout men (*n* = 71)
Age (year)	44.12 ± 10.27	34.87 ± 11.36^**^	42.39 ± 11.95^##^
Body mass index (kg/m^2^)	23.32 ± 3.46	26.80 ± 5.46^**^	26.65 ± 3.38^**^
AST (units/L)	19.57 ± 9.00	29.10 ± 26.35^**^	20,24 ± 5.11^##^
ALT (units/L)	20.69 ± 15.81	42.64 ± 36.70^**^	27.51 ± 13.23^**,##^
Glucose (mmol/L)	4.31 ± 0.58	4.90 ± 1.08^**^	5.56 ± 0.81^**,##^
Triglyceride (mmol/L)	1.15 ± 0.58	2.14 ± 1.36^**^	2.09 ± 1.16^**^
Total cholesterol (mmol/L)	4.23 ± 0.73	4.49 ± 1.07	4.91 ± 0.90^**^
Serum urate (μmol/L)	238.48 ± 36.15	482.47 ± 70.34^**^	517.04 ± 81.16^**^
Systolic blood pressure (mmHg)	118.50 ± 11.75	130.19 ± 11.37^**^	131.52 ± 13.6^**^
Diastolic blood pressure (mmHg)	72.94 ± 8.18	80.48 ± 8.77^**^	81.08 ± 11.89^**^

AST, aspartate transaminase; ALT, alanine aminotransferase. Values are the mean ± SD. ^*^P < 0.05 versus normal controls. ^#^*P* < 0.05 gout patients versus HU patients.

### FAs promote hypoxanthine transport from the blood to the liver for urate biosynthesis in a high fat-induced HU mouse model

**Figure 2:**
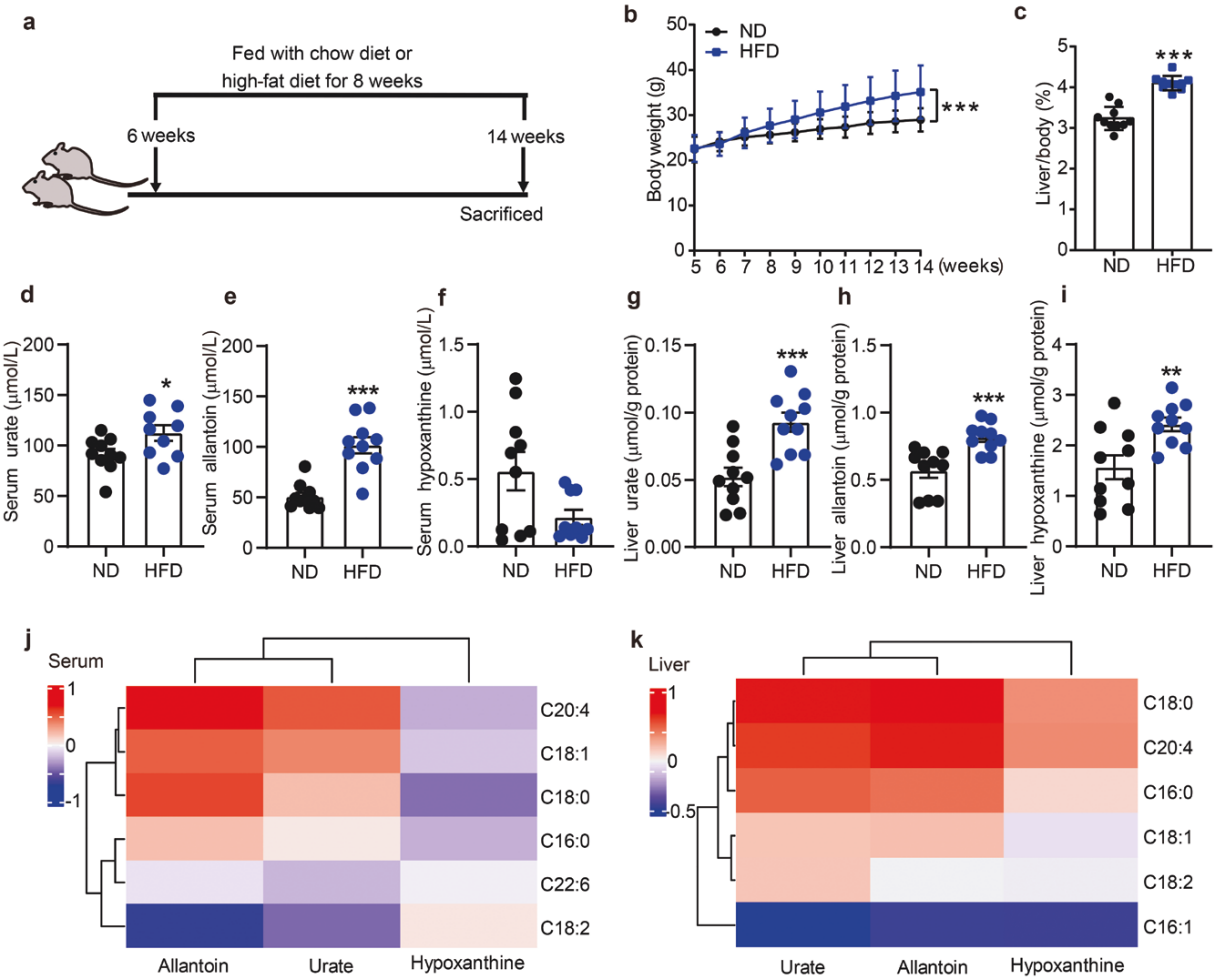
HFD-induced HU mouse model largely recapitulates the correlations of FA levels and major purine metabolites in liver tissue and serum. (a) Schematic diagram of HFD-induced HU mouse model. (b) HFD-induced HU mice have significantly increased body weight during the experimental period. (C) Liver/body weight ratio at 14 weeks (*n* = 10). (d–f) Urate (d), allantoin (e), and hypoxanthine (f) in mouse serum. (g–i) Urate (g), allantoin (h), and hypoxanthine (i) in mouse liver. (j) Heatmap analysis of purine metabolites relative to FAs in mouse serum (*n* = 20). (k) Heatmap analysis of purine metabolites relative to FAs in mouse liver (*n* = 20). ^*^P < 0.05, ^**^P < 0.01, ^***^P < 0.001 by unpaired Mann–Whitney test (f and h) and two-tailed unpaired Student’s *t*-test (b–e, g, and i).

**Figure 3:**
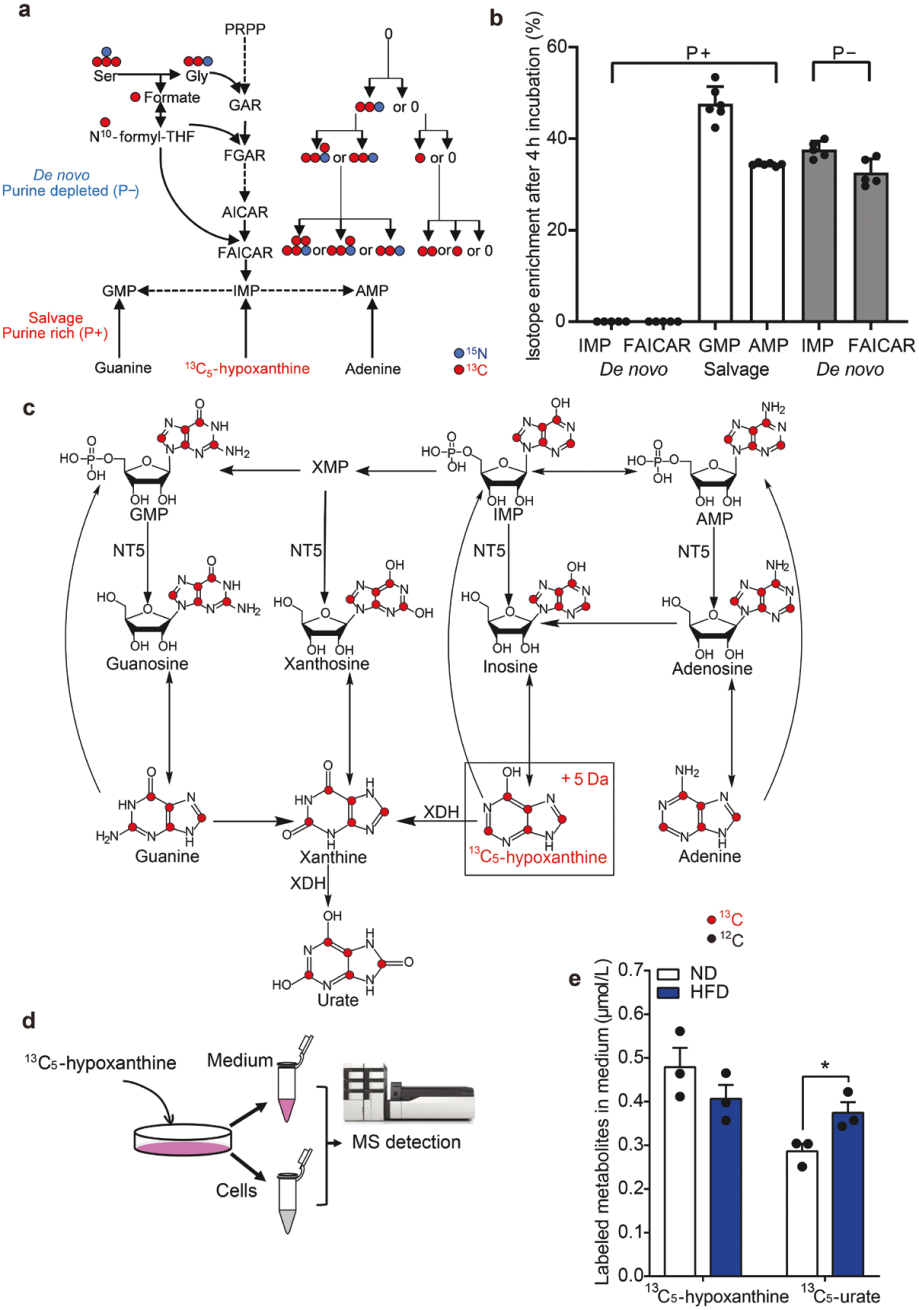
Stable isotope labeled metabolite assay to study purine metabolic pathways. (a) Schematic diagram of purine *de novo* synthesis pathway and salvage pathway with stable isotope labeling under the conditions of purine-rich (P^+^) or purine-depleted (P^−^). The blue dots denote ^15^N, and red dots represent ^13^C. The *de novo* synthesis pathway starts with [^13^C_3_,^15^N]-serine and imparts additional molecular weight into the whole purine pathway through three different steps. There are five possible isotope labeling forms in the end nucleotides, from +1 to +5. (b) The abundance of purine intermediates for P^+^ or P^−^ media is represented by [^13^C_3_,^15^N]-serine incorporation in the products of IMP/FAICAR or ^13^C_5_-hypoxanthine incorporation in AMP/GMP (*n* = 6). (c) ^13^C_5_-hypoxanthine is used to track the isotope label flow into the salvage pathway, imparting a +5 Da mass increment to purine metabolites along the pathway. (d) Schematic diagram of ^13^C_5_-hypoxanthine-derived isotope tracing cell assay. (e) Quantitative results of ^13^C_5_-urate and ^13^C_5_-hypoxanthine in the media after 4 h labeling of mouse hepatocytes (all groups are treated with potassium oxonate to inhibit urate oxidation). *n* = 3. ^*^P < 0.05, ^**^P < 0.01, ^***^P < 0.001 by two-tailed unpaired Student’s *t*-test (e).

To further explore the role of FAs in urate production in the context of HU, we fed mice with a high-fat diet (HFD) or a control normal diet (ND) for 8 weeks (Fig. 2a). Mice in the HFD group had significantly higher body weight and ratios of liver/body weight (Fig. 2b and c). Serum urate and allantoin levels were also significantly increased, whereas hypoxanthine levels were decreased (Fig. 2d–f) compared with the ND group. Notably, the levels of urate, allantoin, and hypoxanthine were all significantly increased in the liver of the HFD group (Fig. 2g–i). The levels of FAs were also significantly higher in the serum and liver tissue in the HFD group compared with the ND group (Supplementary Fig. S3a and b). Similar to humans, the levels of FAs in the serum were positively correlated with urate level but negatively correlated with hypoxanthine level in HFD-fed mice (Fig. 2j), whereas FA levels in the liver tissue were positively correlated with the levels of urate and hypoxanthine (Fig. 2k). These data suggest that FAs in the HFD subjects could increase hepatic urate synthesis with resultant HU by promoting hypoxanthine transport from the circulation, and subsequent transformation of hypoxanthine to xanthine and xanthine to urate by XDH. To test this hypothesis, we developed a metabolic flux assay to track the metabolites in the purine salvage pathway and DNPB pathway using ^13^C_5_-hypoxanthine and [^13^C_3_,^15^N]-serine, respectively (Fig. 3a).

Consistent with previous study, we found Huh7 cells showing significant *de novo* synthesis upon purine depletion (P^−^), indicated by a higher abundance of [^13^C_3_,^15^N]-serine incorporation in the DNPB intermediate 5-formamidoimidazole-4-carboxamide ribonucleotide (FAICAR) and inosine 5ʹ-monophosphate (IMP) (Fig. 3b). When cultured in purine-rich (P^+^) media, however, the cells predominantly demonstrated activation of salvage pathway, quantified by ^13^C_5_-hypoxanthine incorporation in adenosine monophosphate (AMP)/guanosine monophosphate (GMP) (Fig. 3b). Based on these results, our *in vitro* metabolic flux assays were conducted in P^+^ media using ^13^C_5_-hypoxanthine to explore the mechanisms, since liver tissue is normally exposed to a purine rich environment either from endogenous breakdown of RNA or dietary sources (Fig. 3c). In the purine salvage pathway, although ^13^C_5_-hypoxanthine is primarily converted into xanthine and urate via XDH, and to allantoin in mice, it also can be incorporated into other purine metabolites (Fig. 3c).

We initially applied this technique to mice and the distribution of ^13^C_5_-purines in various mouse tissues was quantified after tail-vein injection with ^13^C_5_-hypoxanthine (Supplementary Fig. S3c). Unexpectedly, the highest concentrations of ^13^C_5_-hypoxanthine were found in white adipose tissue, followed by the ileum and the kidney, whereas the liver had minimal levels of ^13^C_5_-hypoxanthine (Supplementary Fig. S3d). Moreover, white adipose tissue and heart tissue had higher levels of total ^13^C_5_-purine (Supplementary Fig. S3e). The ^13^C_5_-urate levels were not detectable in any mouse tissues, likely due to the low levels of ^13^C_5_-hypoxanthine used in our preliminary studies. However, the amounts of unlabeled allantoin (Supplementary Fig. S3f) and urate (Supplementary Fig. S3g) were higher in the jejunum, ileum, and white adipose tissue compared with the controls, consistent with previously reported XOR levels [24]. These results suggest that mouse tissues other than the liver have an extensive metabolic capacity for hypoxanthine, which differs from human purine metabolism [25, 26].

Next, we tested our hypothesis in primary mouse hepatocytes using ^13^C_5_-labeled hypoxanthine after optimizing the incubation time (Fig. 3d; Supplementary Fig. S4). To mimic human hepatocytes, the potassium oxonate was used in both groups to inhibit urate oxidation. We then detected the metabolites in media after labeling and found that the HFD group had lower levels of ^13^C_5_-hypoxanthine but higher levels of ^13^C_5_-urate compared with the ND group (Fig. 3e), consistent with our observations in human serum samples. Therefore, we compared the ^13^C_5_-hypoxanthine consumption and the derived ^13^C_5_-purine metabolites, and found that the majority of ^13^C_5_-hypoxanthine was transformed into ^13^C_5_-urate (Supplementary Fig. S4b and c), with 60% and 81% of ^13^C_5_-urate in the ND and HFD groups, respectively, suggesting that HFD promotes urate production primarily through the uptake of hypoxanthine from the circulation into the liver *via* the purine salvage pathway.

### FA-induced urate biosynthesis is correlated with XDH and 5ʹ-nucleotidase II (NT5C2) levels

**Figure 4:**
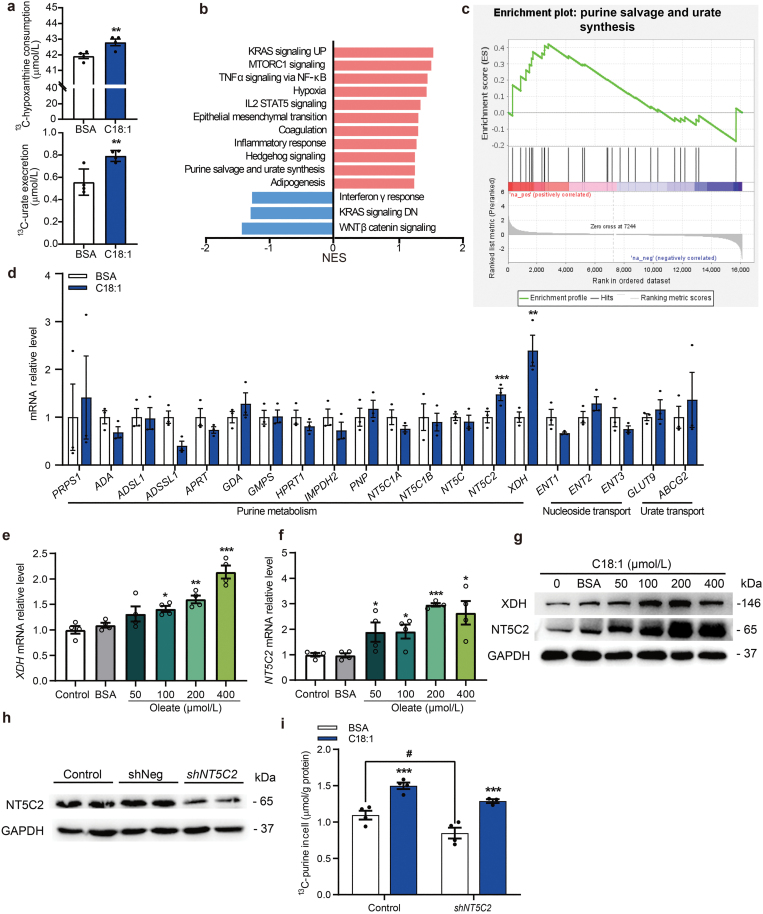
FA-induced hypoxanthine consumption and urate synthesis are associated with XDH and NT5C2 in human Huh7 cells. Sequences of human primers used for qPCR analysis are shown in Supplementary Table S1. (a) Oleate treatment increases ^13^C_5_-hypoxanthine consumption (depleting ^13^C_5_-hypoxanthine from the media) and ^13^C_5_-urate excretion in cell media (*n* = 4). (b) Gene set enrichment analysis (GSEA) using gene expression values of the oleate-treated group (*n* = 3) compared with the BSA-treated group (*n* = 3) against the Hallmark gene sets, showing significantly changed signaling pathways. Gene lists for signaling pathways are shown in Supplementary Table S2. NES, normalized enrichment score. (c) GSEA enrichment plot showing significant enrichment of purine salvage and urate synthesis pathway. (d) The mRNA levels of genes involved in purine metabolism, nucleoside transport, and urate transport with oleate treatment. (e and f) The mRNA levels of *XDH* (e) and *NT5C2* (f) are increased with oleate treatment in a dose-dependent manner. (g) The protein levels of NT5C2 and XDH increased in a dose-dependent manner with oleate treatment. (h) Validation of NT5C2 expression knockdown by Western blot analysis. (i) Purine metabolites are significantly decreased after *NT5C2* knockdown. ^13^C-purine in the cell is the sum of all labeled purine metabolites in cell as shown in Fig. 3c (*n* = 4). ^*^P < 0.05, ^**^P < 0.01, ^***^P < 0.001 by two-tailed unpaired Student’s *t*-test (a and d–f). ^#^*P *< 0.05, ^##^*P *< 0.01, ^###^*P *< 0.001 by two-way-ANOVA (i).

We then examined the mechanism by which HFD or FA metabolism promotes hypoxanthine transport and subsequent urate biosynthesis using the liver cancer cell line Huh7. This cell line had a higher level of XDH expression than other hepatocyte lines although far less than that in the human and mouse livers (Supplementary Fig. S5a–d). To mimic the high-fat environment in cell models, we compared the effects of treatments with different FAs. We found that palmitate, stearate, and oleate treatments all enhanced the consumption of hypoxanthine in cells (Supplementary Fig. S5e). We chose oleate acid (C18:1) as a representative of FAs with consideration of the toxicity of different FAs (Supplementary Fig. S5f). After optimizing the concentrations and treatment time with oleate (Supplementary Fig. S5g and h), we performed metabolic flux assays and found that oleate treatment significantly increased ^13^C_5_-hypoxanthine consumption and ^13^C_5_-purine synthesis in a dose-dependent manner (Supplementary Fig. S5i and j). The derived ^13^C_5_-purine metabolites at different time points with oleate incubation were shown in Supplementary Fig. S6. Under our optimized conditions, we found that oleate treatment significantly increased ^13^C_5_-hypoxanthine consumption and ^13^C_5_-urate secretion (Fig. 4a). These findings were consistent with our observations in the human samples and mouse models.

To explore the mechanisms by which FAs increase hypoxanthine consumption and urate production, we then performed RNA sequence (RNA-seq) after oleate treatment and found that the purine salvage and urate synthesis pathway was among the significantly upregulated pathways (Fig. 4b and c). Next, we surveyed all the genes involved in purine metabolism, nucleoside transport, and urate transport after oleate treatment with quantitative PCR (qPCR), and observed that the expression of *XDH* and *NT5C2* were significantly increased (Fig. 4d). NT5C2 is the enzyme responsible for catalyzing hydrolysis of IMP and GMP [27]. Furthermore, both the mRNA levels (Fig. 4e and f) and protein levels (Fig. 4g) of NT5C2 and XDH increased in a dose-dependent manner with oleate treatment. Knockdown of *NT5C2* led to a significant decrease in intracellular ^13^C_5_-derivatives (Fig. 4h and i). Notably, intracellular ^13^C_5_-derivatives in oleate-treated group were significantly higher than those in the control group (Fig. 4i). Collectively, all these results suggested that FAs may directly promote the consumption of hypoxanthine and urate production by upregulation of NT5C2 and XDH.

### FAO-induced hypoxia-inducible factor 1 alpha (HIF-1α) activation facilitates the transport of hypoxanthine and urate synthesis by upregulating NT5C2 and XDH

**Figure 5:**
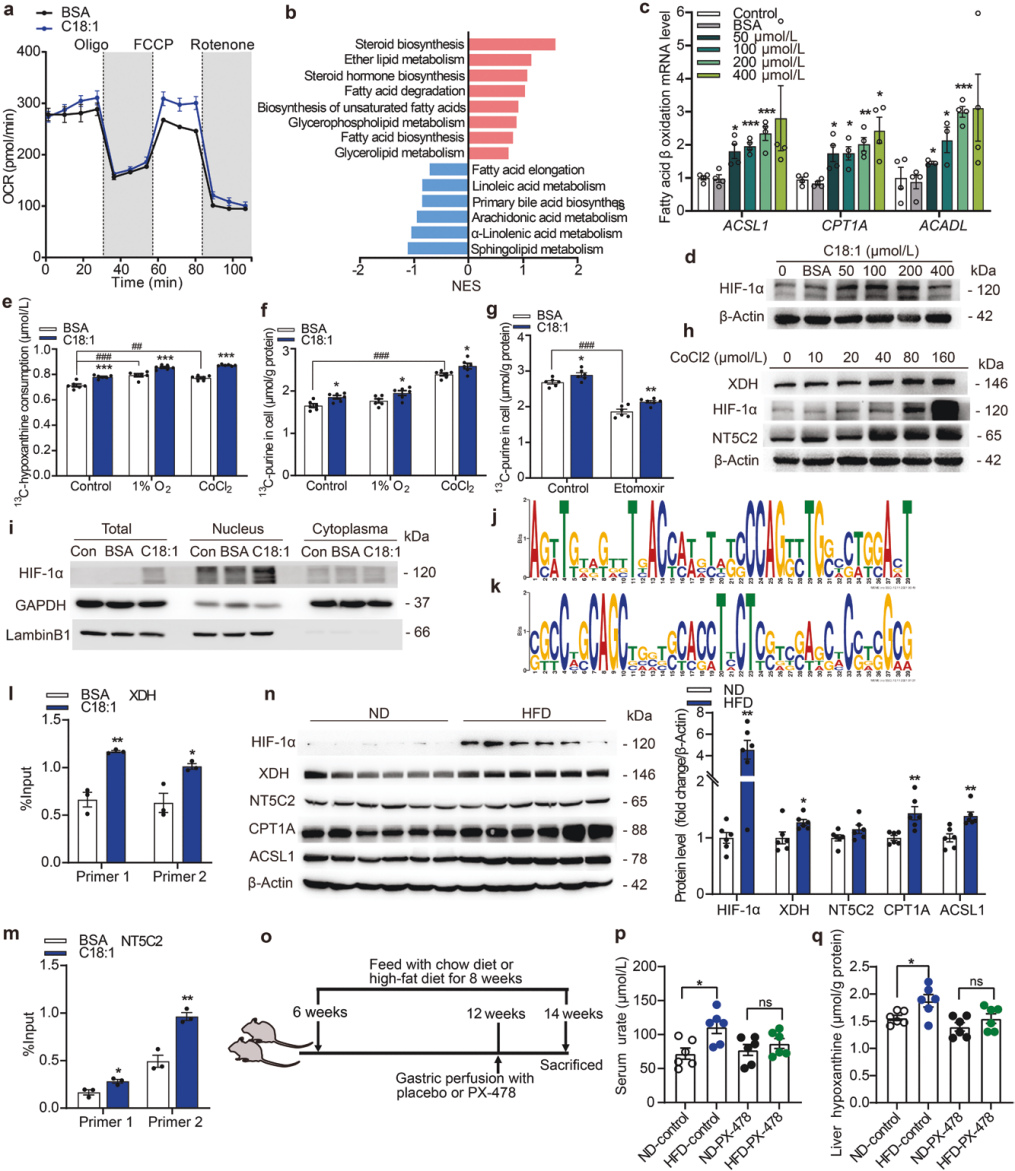
FAO-induced HIF-1α activation upregulates NT5C2/XDH expressions. (a) Oleate treatment increases oxygen consumption rate due to mitochondrial β-oxidation (*n* = 5). (b) GSEA using gene expression values of oleate-treated group (*n* = 3) compared with BSA-treated group (*n* = 3) against the lipid metabolism gene sets in KEGG, showing significantly changed signaling pathways. Gene lists for signaling pathways are shown in Supplementary Table S3. (c) The mRNA levels of *CPT1A*, *ACSL1*, and *ACADL* increase with oleate treatment in a dose-dependent manner. (d) The protein levels of HIF-1α increase in a dose-dependent manner with oleate treatment. (e and f) Hypoxia under 1% O_2_ or CoCl_2_ treatment stimulates hypoxanthine uptake (e) and purine metabolism (f) using ^13^C_5_-hypoxanthine as tracer (*n* = 6). (g) Effects of FA β-oxidation inhibition by etomoxir on ^13^C_5_-metabolites in the cells (*n* = 6). (h) The protein levels of HIF-1α, XDH, and NT5C2 increase in a dose-dependent manner with CoCl_2_ treatment. (I) Oleate treatment promotes HIF-1α entering the nucleus. (j and k) The HIF-1α-binding motifs enriched in *XDH* promoter (j) and *NT5C2* promoter (k) are predicted by MEME. (l and m) ChIP-qPCR assay is performed to confirm that HIF-1α binds to the promoter region of *XDH* (l) in (−1776/−1631) and (−1429/−1327), and *NT5C2* (m) in (− 555/−357) and (− 398/−246). (n) The proteins involved in the purine salvage pathway increase in HFD-stimulated mouse liver. (o) Experimental design of mouse model treated with control vehicle or PX-478 (5 mg/kg body weight, *per os*, every 2 days). (p) Urate levels in mouse serum after PX-478 treatment. (q) Hypoxanthine levels in mouse liver after PX-478 treatment (*n* = 6). ^*^P < 0.05, ^**^P < 0.01, ^***^P < 0.001 by two-tailed unpaired Student’s *t*-test (c, l−n, and p−q). ^#^*P *< 0.05, ^##^*P *< 0.01, ^###^*P *< 0.001 by two-way-ANOVA (e−g).

Since FA β-oxidation may lead to HIF-1α activation [28], hypoxia-induced XDH activity has also been observed in rat pulmonary microvascular endothelial cells (RPMEC) [29]. Consistently, our RNA-seq results showed that hypoxia was also a significantly altered pathway with oleate treatment (Fig. 4b; Supplementary Fig. S7a). We then tested the hypothesis that HIF-1α was involved in upregulating *NT5C2* and *XDH* expression for hepatic urate biosynthesis. We observed that oleate treatment significantly increased the oxygen consumption rate, consistent with an elevated FA β-oxidation in the mitochondria (Fig. 5a). Our RNA-seq analysis, with FA degradation being the enriched pathway with oleate treatment (Fig. 5b; Supplementary Fig. S7b), did not enrich pathways related to FA transporters. Indeed, oleate treatment dose-dependently upregulated the mRNA levels of FA β-oxidation related genes, including long-chain acyl-CoA dehydrogenase (*ACADL*), acyl-CoA synthetase long-chain family member 1 (*ACSL1*), and carnitine palmitoyl transferase1a (*CPT1A*) (Fig. 5c). Besides, the protein levels of HIF-1α also increased in a dose-dependent manner with oleate treatment (Fig. 5d). To further investigate the role of HIF-1α in hypoxanthine transport and urate biosynthesis, we performed tracing experiments under hypoxia conditions with 1% O_2_ or CoCl_2_ treatment. Consistently, HIF-1α activation led to a significant increase in ^13^C_5_-hypoxanthine uptake and intracellular ^13^C_5_-derivatives, which were further enhanced with C18:1 treatment (Fig. 5e and f). On the other hand, inhibition of FA β-oxidation by etomoxir treatment led to a significant decrease of intracellular ^13^C_5_-derivatives (Fig. 5g).

Next, we examined whether HIF-1α regulates XDH and NT5C2 expressions. HIF-1α activation with CoCl_2_ treatment dose-dependently increased the protein levels of XDH and NT5C2 (Fig. 5h). Furthermore, oleate treatment led to a higher nuclear level of HIF-1α (Fig. 5i), suggesting that more HIF-1α entered the nucleus to upregulate the expressions of XDH and NT5C2. We then searched for potential HIF-1α binding sites and motifs in the promoters of *XDH* and *NT5C2* genes using an online tool MEME (Multiple Expectation Maximization for Motif Elicitation) [30, 31] (Fig. 5j and k), and these tentative HIF-1α binding sites in *NT5C2* and *XDH* stimulated by FAO were confirmed by chromatin immunoprecipitation (ChIP)-qPCR (Fig. 5l and m). Importantly, HIF-1α, XDH, and two FA β-oxidation-related proteins, CPT1A and ACSL1, were significantly upregulated in the livers of mice fed with HFD compared to ND (Fig. 5n).

To further investigate the role of HIF-1α activation in hepatic urate synthesis *in vivo*, we treated HFD-induced HU mouse model with HIF1-α inhibitor PX-478 (Fig. 5o; Supplementary Fig. S8). We found that after treatment with PX-478 for 2 weeks, the elevated levels of serum urate induced by HFD were completely diminished (Fig. 5p). Similar trend was observed for liver hypoxanthine levels (Fig. 5q). The body weight of the mice with PX-478 treatment and other purine metabolites in the serum and the liver are shown in Supplementary Fig. S8.

Together, these data suggest that FA β-oxidation-induced HIF-1α activation leads to upregulation of NT5C2/XDH to facilitate the transport of hypoxanthine and subsequent urate biosynthesis.

### FAO promotes hepatic urate synthesis in humans through the HIF-1α-NT5C2/XDH pathways

**Figure 6:**
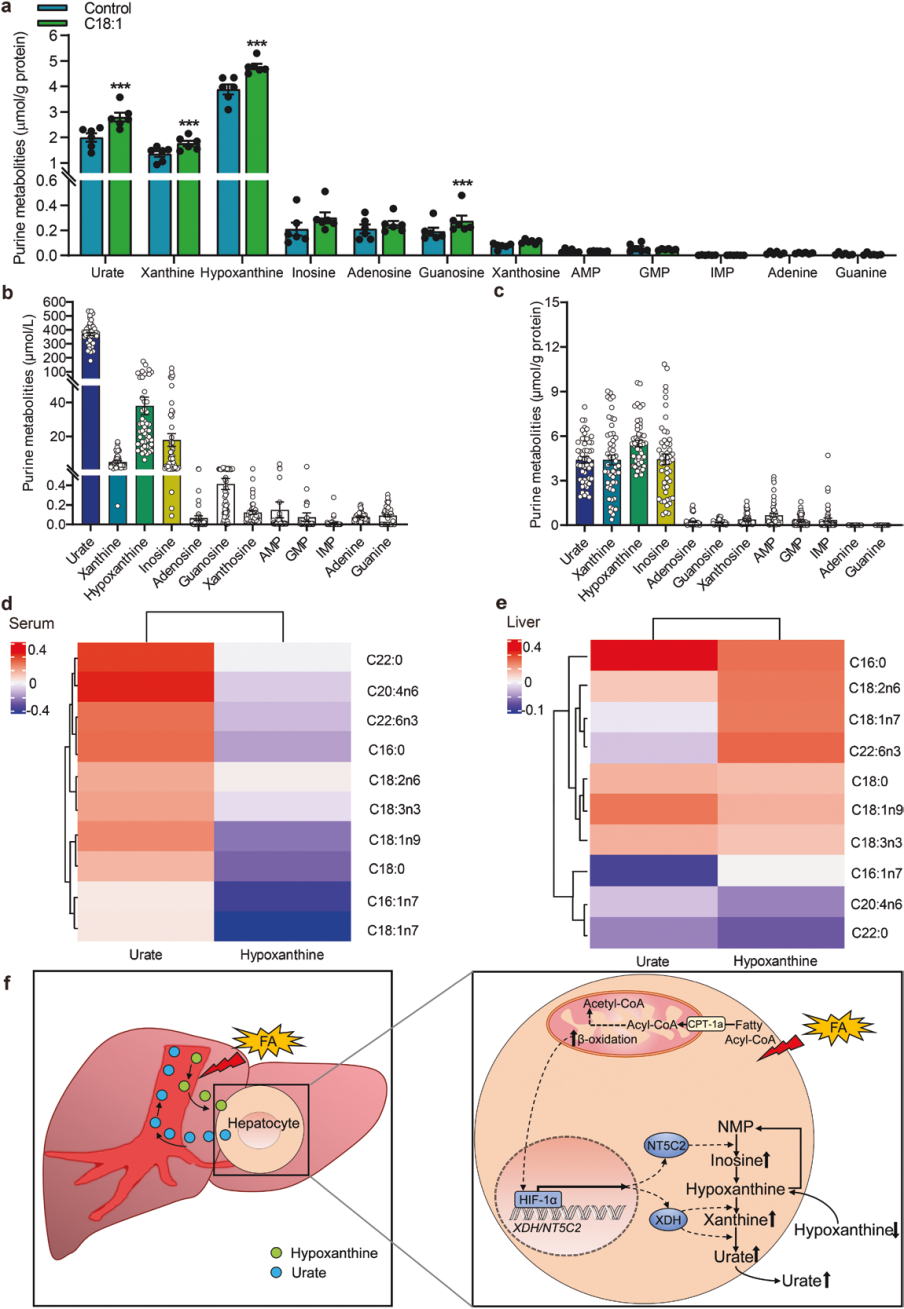
FAs and the purine salvage pathway in human hepatocytes and the liver. (a) Quantitative results of purine metabolites in human hepatocytes after oleate treatment (*n* = 6). (b and c) Quantitative purine metabolites in paired serum (b) and livers (c) (*n* = 50). (d and e) Heatmap analysis of purine metabolites relative to FAs in paired serum (d) and liver samples (e) (*n* = 50). (f) Working model. FAs promote hepatic urate synthesis by upregulating purine salvage pathway and leading to HU. FA β-oxidation activates HIF-1α and subsequently upregulates the expression of *NT5C2* and *XDH*, which catalyzes hypoxanthine metabolism to NMP (nucleoside monophosphates), xanthine, and urate. ^*^P < 0.05, ^**^P < 0.01, ^***^P < 0.001 by two-tailed unpaired Student’s *t*-test (a).

Due to the difference in purine metabolism in rodents and humans, we next examined the relevance of FAO-induced HIF-1α activation in urate biosynthesis in humans. Firstly, we isolated human hepatocytes to perform isotope labeling test to confirm our findings. The unlabeled purine metabolites in human hepatocytes showed that hypoxanthine and urate increased significantly with oleate treatment (Fig. 6a) and the ^13^C_5_-metabolites also increased significantly (Supplementary Fig. S9a). The purine metabolites in media showed that the consumption of ^12^C-hypoxanthine and ^13^C_5_-hypoxanthine and export of ^12^C-urate and ^13^C_5_-xanthine (Supplementary Fig. S9b and c) increased after oleate treatment.

To provide further evidence to support our hypothesis, we performed targeted metabolomic analysis of purine metabolites and FAs in 50 pairs of serum and liver tissues from healthy male liver transplant donors. The clinical characteristics of the participants are shown in Table 2. We detected 12 major purine metabolites in human serum and liver tissues (Fig. 6b and c). Interestingly, urate, hypoxanthine, inosine, and xanthine represented the top four most abundant purine metabolites in human serum, with urate as the most predominant metabolite. However, in human liver tissues, these four metabolites were major metabolites but with comparable levels. Furthermore, the levels of urate in the serum appeared positively correlated with that in the liver, whereas hypoxanthine showed a reverse trend (Supplementary Fig. S9d and e). The levels of FAs in human serum and liver were also measured (Supplementary Fig. S9f and g). The levels of urate were positively correlated with most FAs in both serum and liver tissues, while hypoxanthine was negatively correlated with FAs in the serum but positively correlated in the liver (Fig. 6d and e). Moreover, we further equally divided the samples based on the levels of TFAs in liver tissues (Supplementary Fig. S9h) and found that the urate levels in the serum and liver tissues of the high TFA group were higher than those in the lower TFA group (Supplementary Fig. S9i), whereas the hypoxanthine levels in the serum of the TFA-high group were significantly decreased compared with the TFA-low group (Supplementary Fig. S9j). The levels of hypoxanthine in the liver and the ratios of hypoxanthine in the liver/serum were higher in the TFA-high group compared with those in the TFA-low group (Supplementary Fig. S9j).

**Table 2.** Clinical and demographic characteristics of liver transplant subjects.

	Entire group (*n* = 50)	1st dichotomousness (*n* = 25)	2nd dichotomousness (*n* = 25)	*P* for trend
Body mass index (kg/m^2^)	23.24 ± 2.38	22.75 ± 2.39	23.72 ± 2.27	0.16
Age (year)	34.42 ± 6.95	34.20 ± 6.67	34.64 ± 7.21	0.83
Glucose (mmol/L)	5.21 ± 0.58	5.12 ± 0.37	5.30 ± 0.71	0.29
Triglyceride (mmol/L)	1.24 ± 0.68	1.03 ± 0.46	1.45 ± 0.78	0.03
Total cholesterol (mmol/L)	4.37 ± 0.81	4.27 ± 0.78	4.48 ± 0.82	0.38
Serum urate (μmol/L)	388.04 ± 79.43	371.58 ± 52.45	404.50 ± 96.57	0.16

Values are the mean ± SD.

Taken together, these data strongly support that FAO promotes hepatic urate synthesis in humans through the HIF-1α-NT5C2/XDH pathways.

## Discussion

In this study, we have discovered a novel mechanism by which FAO facilitates hepatic urate biosynthesis by activating HIF-1α, leading to the upregulation of *NT5C2* and *XDH* expression to enhance the consumption of hypoxanthine, thus accumulating the transport of hypoxanthine by equilibrative nucleoside transporter [32] from the circulation to the liver for subsequent urate synthesis through purine salvage pathway (Fig. 6f). The results directly linked dyslipidemia and HU.

The unexpected pattern of hypoxanthine in our targeted metabolomic analysis of a human cohort comprised of people with HU and gout prompted us to examine the underlying mechanisms regulating urate biosynthesis in the liver. It has long been established that urate can be generated from the DNPB pathway and the purine nucleotide salvage process [8], but the contributions of these two pathways to HU are less clear [33], especially the purine salvage pathway [34]. The majority of HU people with endogenous urate overproduction appear to be due to the salvage pathway [8]. In this study, we identified an under-appreciated role of hypoxanthine in urate production. Our data show that hypoxanthine is the most abundant purine metabolite in the human liver and the second most abundant metabolite in the serum (Fig. 6b and c). Hypoxanthine in human blood circulation mainly comes from endogenous purine metabolism and exogenous diet, but the contributions of these two sources remain poorly defined. Early studies found that during short-term exhaustive exercise, skeletal muscle excretes a large amount of hypoxanthine into the blood, which can be taken up by the liver to synthesize urate [35]. Another study found that human adipose tissue can secrete hypoxanthine and the secretion increases in local hypoxia [36], but the contributions of different tissues and organs to the hypoxanthine pool are still unclear. Similarly, exogenous hypoxanthine comes from the diet, and different foods contain different levels of hypoxanthine. A prior investigation involving human subjects observed the impact on blood urate levels following the consumption of equimolar doses of various purines. The study revealed a notable elevation in urate levels specifically in response to hypoxanthine, with a more pronounced effect observed in individuals afflicted with HU and gout [37].

We observed that hypoxanthine levels were negatively correlated with TFAs in human serum, but positively in human liver tissue from liver transplant subjects. Similar observations were made in the HU mouse model. Further study uncovered HIF-1α as a critical factor in regulating hepatic urate biosynthesis in the context of FA β-oxidation. Human serum urate increases significantly in the setting of decreased oxygen saturation at high altitudes [38]. Interestingly, sleep apnea has been associated with HU and incident gout through HIF-1α activation [39, 40]. HIF-1α activation is involved in alcohol-induced liver injury and steatosis [41], whereas HU induces liver injury by upregulating HIF-1α and inhibiting arginine biosynthesis in mice [42]. It is well-documented that the human liver of NAFLD is often accompanied by increased FA β-oxidation [28] and HIF-1α activation [43], especially in the early stage of NAFLD, hepatosteatosis [19, 20], but the precise mechanism by which HIF-1α is activated remains uncertain at normoxia. In this study, we found that HIF-1α activation enhances hypoxanthine transport from the blood to the liver tissue for subsequent urate synthesis.

HIF-1α was significantly upregulated in the liver tissue of HFD-induced HU mouse model, along with upregulation of XDH and NT5C2 (Fig. 5n). Our results are consistent with previous reports in hypoxia-induced alterations of purine metabolism in different tissues. During hypoxia, ATP is metabolized along enzymatically regulated cascades for intracellular energy production, while AMP is accumulated in other ways and degraded to adenosine in the blood to be further converted to hypoxanthine [44]. Hypoxanthine then accumulates in the setting of hypoxia, as its degradation to xanthine/urate and conversion to IMP are limited due to oxygen deficiency [45]. Moreover, the secretion of urate is enhanced in the adipose tissues of obese mice [24], and local hypoxia upregulates XDH activity in adipocytes. Increased *XDH* mRNA and protein levels have been confirmed in bovine arterial endothelial cells [46] and the liver of the NAFLD mouse model [21].

Our study uncovered a novel role of *NT5C2* in FA-induced hepatic urate synthesis as a target gene downstream of HIF-1α. Previous studies primarily focused on NT5C2 in the AMP-activated protein kinase (AMPK) pathway. For example, genetic deletion of *NT5C2* in mice reduces body gain and insulin resistance induced by HFD feeding [47]. Interestingly, *NT5C2* KO mice exhibit lower levels of urate on HFD feeding compared to wild-type mice, but the mechanisms remain elusive [47]. However, gain-of-function relapse-associated mutant forms of *NT5C2* increase downstream purine secretion in acute lymphoblastic leukemia cells, including urate and xanthine [48]. Here, our studies found that *NT5C2* is a target gene of HIF-1α and its upregulation with *XDH* synergistically promotes urate biosynthesis in the liver.

Differences in the purine metabolic pathways between human and mouse models pose a tremendous challenge to studying urate biosynthesis in the human context of HU and gout [25, 26, 49]. The application of techniques such as metabolomics and metabolic flux analysis in human samples or human-derived cell models may provide unprecedented insights into studying the pathological basis of HU and gout [50]. A recent study from our group systematically profiled the metabolomic differences in people with HU and gout compared to normauricemia controls using non-targeted metabolomics [23]. In the current study, targeted metabolomic analysis in the same cohorts revealed a unique pattern of hypoxathine, which led us to further examine the underlying mechanisms of FA metabolism and purine metabolism in HU and gout. The stable isotope labeled metabolic flux analysis based on glucose has been widely used to study metabolic reprogramming in cancer [51]. In this study, we developed targeted metabolic flux assays to study purine metabolism in HU and gout. Due to that the incorporation of ^13^C_5_-hypoxanthine into purine metabolites by salvage pathway does not change +5 Da labeling, we can measure the absolute levels of metabolites with labeling along the pathway rather than the ratio. We found that ^13^C_5_-urate excretion in HFD mouse hepatocytes was increased after oxonate treatment, with similar results observed in human hepatocytes (Fig. 6a). An unexpected finding was that in addition to the liver, other tissues in mice make significant contributions to whole-body urate generation through purine salvage pathways via uptake of hypoxanthine (Supplementary Fig. S3d–g), presumably through enhanced XDH activity [24]. These data exemplify the challenges of studying urate production in mouse models. A recent genome-wide association study of over 2.6 million individuals identified XDH activity in the prostate in the progression from HU to gout [52], but whether the human prostate produces urate and  to what extent it has an effect on whole-body blood urate levels remain to be determined. Further studies are warranted to clarify the roles of purine salvage pathways in different human tissues in the context of urate production, relative to the DNPB pathway.

In summary, we found that FAO directly increases serum urate levels by activating HIF-1α-NT5C2/XDH in the liver to facilitate the purine nucleotide salvage pathway. Our study provides novel evidence linking HU to other metabolic diseases associated with elevated levels of FAs, such as obesity, hyperlipidemia, and NAFLD. This study has further consolidated the role of HIF-1α activation under hypoxic conditions in the pathogenesis of HU, such as sleep apnea and high altitude. These findings have laid the foundation for exploring potential new therapeutic targets for people with HU and disorders of lipid metabolism. Although our current studies were carried out in male mice and human participants due to a much higher incidence of HU and gout in men, similar mechanisms presumably operate in females. Future studies are warranted to investigate the role of sex hormones in this pathway.

## Materials and methods

### Study participants

A total of 241 male participants were enrolled as we described before [23]. Briefly, the participants included outpatients and participants enrolled at the dedicated Gout Clinic of the Affiliated Hospital of Qingdao University. Since the prevalence of HU and gout in China is much higher in men than women, we only enrolled male participants in this study to minimize the hormonal effects. The diagnostic criteria for HU and gout were based on rules [53]. Participants were not permitted to take drugs affecting the serum urate level in the 2-week washout period prior to enrollment. If a gout flare occurred during the period, immediate medical treatment was given and patients were excluded from this study. Participants with any known metabolic diseases were also excluded. A total of 50 healthy male liver transplant donors were enrolled from Renji Hospital Affiliated to the Medical College of Shanghai Jiao Tong University. The inclusion criteria for healthy liver donors include: (i) aged 18 years or older; (ii) available to obtain serum samples before surgery; (iii) available to obtain normal liver tissues during surgery. The exclusion criteria contain: (i) missing clinical information; (ii) fatty liver and other metabolic diseases. The written informed consent was obtained from all participants. All the clinical studies were approved by the ethics committee in the Hospitals Affiliated with Qingdao University, Qingdao (ChiCTR1900022981), and Renji Hospital Affiliated with Shanghai Jiao Tong University School of Medicine, Shanghai, China (KY2021-063-B).

### Animals

Male C57BL/6J mice were housed in a pathogen-free facility under a normal light cycle (light on 8:00−20:00). Mice (5 weeks old) were purchased from Shanghai Slack Laboratory Animal Corp. Ltd. Mice were allowed 7 days to acclimate to the facilities before experiments and were randomly divided into two groups and treated for 8 weeks. The control group was fed with an ND (P1101F, SLACOM), which contains kcal of 13% from fat, 62% from carbohydrates, and 25% from protein. The test group was fed with an HFD (D12492, Research Diets, New Brunswick, NJ), which contains kcal of 60% from fat, 20% from carbohydrates, and 20% from protein.

For the HIF-1α effect assessment, the mice were randomly divided into 4 groups (*n* = 6 mice in each group). After 6 weeks of diet treatment, the mice were administered intragastric administration once every two days for 2 weeks with the diet remaining unchanged. The control group received a placebo (0.5% sodium carboxymethyl cellulose), and the treatment group received PX-478 (5 mg/kg body weight). All animal experiments conformed to the National Institutes of Health (NIH) guidelines for the care and use of laboratory animals approved by the Institutional Ethics Committee on Animal Care at the Shanghai Institute of Nutrition and Health, Chinese Academy of Sciences.

### Human sample collection

The venous blood samples were obtained from participants after overnight fasting. The serum samples were obtained after blood clotting at room temperature for 30 min and then centrifuged at 3000 *g* for 10 min. The serum samples were stored at −80°C before analysis. The normal liver tissues were obtained from 50 healthy male liver donors during liver transplantation. Tissue samples were stored at −80°C before use.

### Mouse sample collection

In the 14th week, the mice from the two groups were sacrificed. Food was removed in the evening at 21:00 prior to sacrifice, and on the day of the experiment, mice were sacrificed around 9:00 a.m.. Blood was collected by heart puncture from isoflurane-anesthetized mice, then kept on ice to coagulate and obtain serum after centrifugation at 1000 *g* at 4°C for 15 min. The liver tissues were collected and cut on a glass plate while kept on top of the ice. All tissues were immediately dropped in liquid nitrogen.

### Primary hepatocyte collection and cell culture

Primary hepatocytes were isolated from male C57BL/6 mice using collagenase perfusion [54], and cultured on collagen-coated plates in high glucose (4.5 g/L) Dulbecco’s modified Eagle’s medium (DMEM) supplemented with 1% penicillin–streptomycin, 100 nmol/L insulin, 100 nmol/L dexamethasone, 10% fetal bovine serum (FBS), and 1% glutamax. Fresh para-cancerous liver tissues were obtained after hepatectomy and immediately digested by collagenase (type I:type IV = 1:1) in an incubator at 37°C [51]. After 30 min, cells were collected by centrifuging at 1000 *g* at 4°C for 5 min. Finally, the cells were seeded in 6-well plates coated with collagen and incubated in DMEM supplemented with 10% FBS, 1% penicillin–streptomycin, 10 ng/mL epidermal growth factor (EGF), 10 μg/mL insulin, and 2 μmol/L hydrocortisone and were cultivated for different experiments. Huh7 cells were purchased from CAS Cell Bank and cultured in DMEM with 10% FBS and 1% penicillin–streptomycin. All cells were cultured in an incubator at 37°C with 5% CO_2_.

### LC-MS sample preparation

Samples were stored at −80°C and thawed on ice for use. For serum samples, 100 μL samples were transferred into a tube for use. For liver tissues, 20 mg samples were weighed into the tube and homogenized with 200 μL water. About 30 μL samples were used to quantify protein concentrations. Soluble metabolite extraction was done by adding −80°C methanol (MeOH, 400 μL) with 10 μmol/L fluorouracil. Samples were vortexed for 10 s, sonicated in a 0°C water bath for 10 min, and incubated at –80°C for 2 h. The supernatant was collected after centrifugation at 16000 *g* at 4°C for 15 min and then dried under N_2_. The residue was reconstituted in 200 μL mobile phase buffer A after centrifugation and then transferred to LC-MS vials for analysis. Quality control was pooled from samples (5 μL of each sample) and used throughout the run of each batch. All samples were randomized during data acquisition.

### LC-MS method

To quantify polar metabolite concentrations, we used a UHPLC system (Nexera UHPLC LC-30A, SHIMADZU Technologies, Japan) coupled to an Electrospray ion source (ESI) mass spectrometer (LCMS-8050, SHIMADZU Technologies, Japan) in positive (+ESI) mode. Samples were eluted on a UPLC column (Amid 100 mm × 2.1 mm, 1.7 μm particle size; part no: 186004801, Waters) at 30°C with a flow rate of 0.2 mL/min. Mobile phase A was ultrapure water containing 0.1% formic acid, and mobile phase B was ultrapure water containing 0.1% formic acid and 20% acetonitrile. Samples were eluted according to the following gradient: 0–0.5 min, 5% B; 0.5–3.5 min, 5% B to 100% B; 3.5–6 min, 100% B; 6–7 min, 100% B to 5% B; 7–10 min, 5% B. The MS was operated in a multiple reaction monitoring (MRM) mode and targeted metabolite transitions were from authentic standards. The interface temperature was set at 300°C while the desolation line and heat block temperatures were set at 250°C and 400°C, respectively. The interface voltage was maintained at 3 kV. Nitrogen was used as nebulizing gas at a flow of 3 L/min while drying gas and heating gas flows (also nitrogen) were maintained at 10 L/min. Argon was used as collision gas at 230 kPa.

### GC-MS for FA measurement

TFAs were extracted as our laboratory published previously [55]. Briefly, 2 mL chloroform:methanol (2:1, v/v) with 20 μg phosphatidylcholine (PC) (21:0) was added to 1 mL homogenates (80 μL serum or 20 mg tissues in 1 mL water). The mixture was shaken for 5 min and then centrifugated at 2000 *g* for 10 min. The chloroform phase was dried under N_2_ gas and then derivatized to form FA methyl esters (FAMEs) via the addition of 2 mL 2% H_2_SO_4_ in methanol and incubation at 80°C for 1 h. Next, 2 mL hexane and 0.5 mL ddH_2_O were added, the hexane layer was dried after centrifugation, and 0.2 mL hexane was added to dissolve FAs. TFAs were analyzed using a select FAME column (SPTM-2560, 0.2 μm, 100 m × 0.25 mm) installed in a Shimadzu QP-2010 Ultra GC-MS with an injection volume of 1 μL (10:1 split ratio). GC-MS parameters were as follows: oven temperature started at 160°C for 1 min, rising to 175°C at 5°C/min with hold time for 3 min, to 210°C at 1°C/min with hold time for 5 min, and to 240°C at 5°C/min with hold time for 20 min. GC-MS interface temperature was set at 250°C and (electron impact) ion source temperature was set at 300°C, with 70 V/60 μA ionization voltage/current. The mass spectrometer was set to scan *m/z* range 40–800, with a 0.1 kV detector. The metabolites were quantified on the basis of total ion count peak area, using standard curves generated from running standards in the same batch of samples.

### Metabolic flux experiments

For labeling experiments, primary hepatocytes were isolated, and cultured for 12 h before labeling. Huh7 cells were changed into the same condition as primary hepatocytes 24 h before tracing. Cells were seeded at a density of approximately 5 × 10^5^ cells per plate in 6-well plates. Labeling medium (with 1 μmol/L [^13^C_5_]-hypoxanthine) was used to replace the unlabeled medium at 0 h. After 4 h of isotope incubation, 100 μL medium was aspirated and 500 μL cold MeOH (containing 10 μmol/L 5-fluorouracil) was added. Cells were washed three times with 1× PBS, and 500 μL cold MeOH (containing 10 μmol/L fluorouracil) was directly added to the culture dish to harvest cells by scraping. In each group, an extra well was prepared to collect proteins as a loading control. Samples were incubated at –80°C for 2 h, and then thawed on ice and centrifuged at 13,000 *g* at 4°C for 15 min to obtain supernatant. The supernatant was dried under N_2_ gas flow and reconstituted in 100 μL mobile phase buffer A, and 2 μL of the extract was used for each LC-MS run.

### ChIP-qPCR analysis

Briefly, oleate and 5% BSA-treated Huh7 cells were fixed with 1.5% formaldehyde for 10 min to cross-link DNA-protein complexes. Genomic DNA was extracted and sheared to 200–900-bp fragments using a sonicator. DNA-protein complexes were immunoprecipitated with indicated antibodies (HIF-1α and IgG) using the SimpleChIP® Plus Enzymatic Chromatin IP Kit (Cell Signaling Technology, cat#9005) according to the manufacturer’s instructions. *XDH* and *NT5C2* promoter primers were listed as follows: for human *XDH*,

Primer 1: 5ʹ-CCAGACATTGCCAAGTGGCTTCT-3ʹ and 5ʹ-CGGAGGTTGCAGTGAGCTGAAAT-3ʹ;

Primer 2: 5ʹ-ATGCCCAGCCAACAACCAGTTT-3ʹ and 5ʹ-AGCCTTGAATCCCTGACAAATGCC-3ʹ;

For human *NT5C2*,

Primer 1: 5ʹ-CGCCGATACTCGTGGATCTCCT-3ʹ and 5ʹ-AGGTGGACGTGACTGTGGCTAA-3ʹ;

Primer 2: 5ʹ-GAGCCGAGGTCAGGTCTGGTTTA-3ʹ and 5ʹ-AGAAGAAGGTGGAGTCGCTGCT-3ʹ.

### RNA-seq of Huh7 cells

RNA-seq of Huh7 cells was performed at Integrated Genomics Operation, BGISEQ. Total RNA was extracted from 5 × 10^5^ cells using Trizol reagent (Invitrogen, 15596018) and quantified by Nanodrop and Qsep-100 (Thermo Fisher Scientific). Samples were barcoded and run on the MGI 2000 platform. The sequencing data was filtered with SOAPnuke [56] to obtain clean reads and stored in FASTQ format, and the raw sequencing reads were aligned to a human reference genome (GCF_000001405.39_GRCh38.p13) using Bowtie2 [57]. The gene expression level was calculated by RSEM (v1.3.1) [58]. Gene set enrichment analysis was conducted by GSEA software (v4.3.2) against the Hallmark or KEGG gene sets [59, 60].

### Statistical analysis

The statistical analyses are described in the figure legends. All data were expressed as mean ± SEM. The tests used included two-tailed unpaired Student’s *t*-test, Kruskal–Wallis test, Mann–Whitney test, one-way ANOVA, and two-way ANOVA. Statistical analyses were performed using Microsoft Excel 2019 and Prism 8 (GraphPad Software). Statistical significance was set at *P* values < 0.05.

## Supplementary Material

loae018_suppl_Supplementary_Figures_Table_S1

loae018_suppl_Supplementary_Table_S2

loae018_suppl_Supplementary_Table_S3

## Data Availability

All data relevant to the study are included in the article or uploaded as supplementary information. The data used to support the findings of this study will be available on request.
