# Supplementary material for: Fatty acid oxidation-induced HIF-1α activation facilitates hepatic urate synthesis through upregulating NT5C2 and XDH
Source: Life Metab. 2024 May 17;3(5):loae018. doi: 10.1093/lifemeta/loae018 (PMC11749550; doi:10.1093/lifemeta/loae018)
Supplement: loae018_suppl_Supplementary_Figures_Table_S1 [file loae018_suppl_Supplementary_Figures_Table_S1.pdf]

Supplementary information

Fatty acid oxidation-induced HIF-1α activation facilitates hepatic urate synthesis through upregulating *NT5C2* and *XDH*

Table of contents

Supplemental Figure S1……………………………………………………………...2

Supplemental Figure S2…………………………………………………………….3

Supplemental Figure S3…………………………………………………………….4

Supplemental Figure S4…………………………………………………………….5

Supplemental Figure S5…………………………………………………………….6

Supplemental Figure S6…………………………………………………………….7

Supplemental Figure S7…………………………………………………………….8

Supplemental Figure S8……………………………………………………………..9

Supplemental Figure S9……………………………………………………………10

Supplemental Note 1…………………………………………………………......12

Supplementary materials and methods……………………….……….…………..13

Supplementary Table S1…………………………………………………………....14

Supplementary Figure S1 Related to Figure 1.

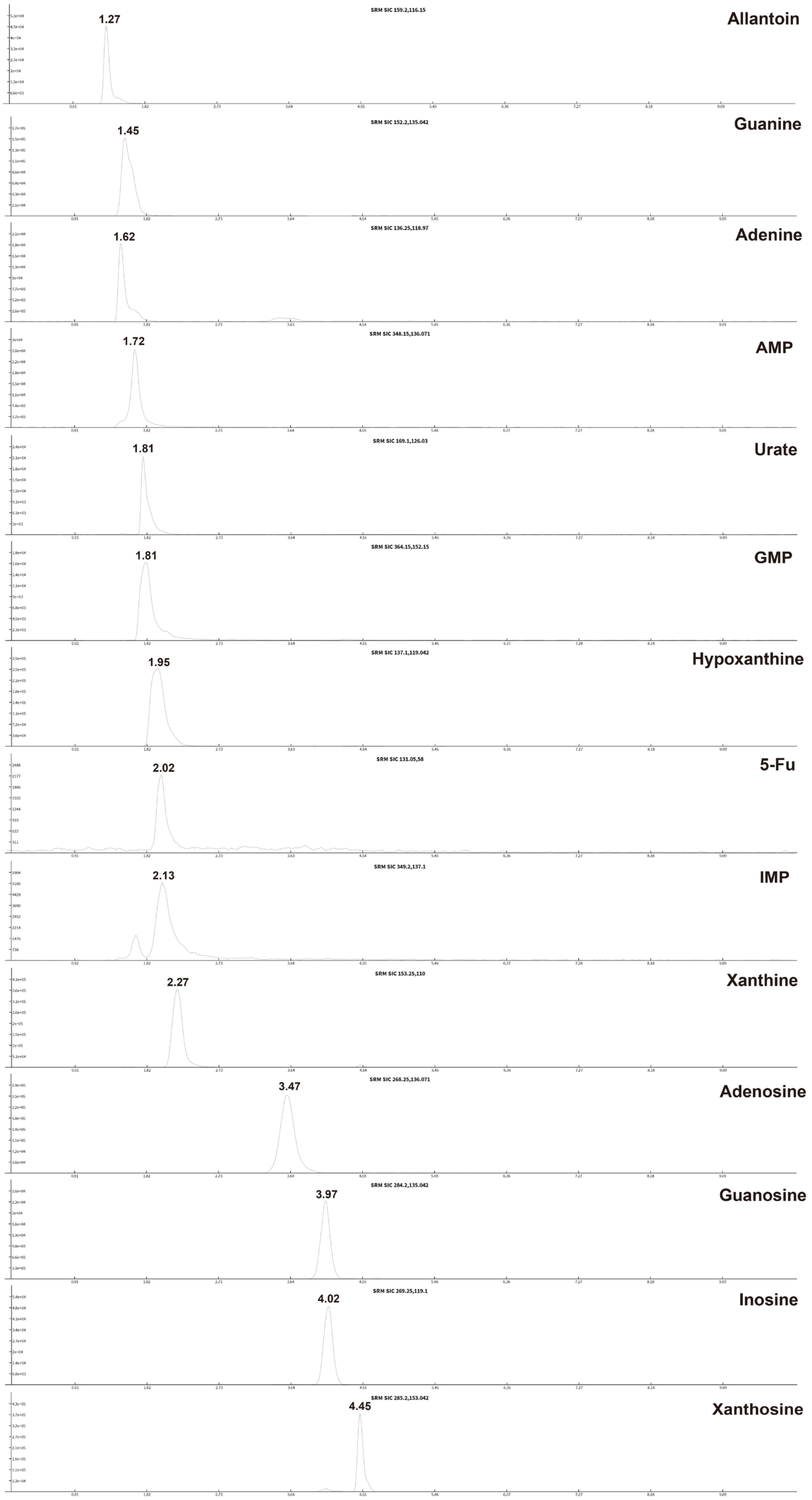

Supplementary Figure S1 The multiple reactions monitoring spectrums of 13 purine standards.

Supplementary Figure S2 Related to Figure 1.

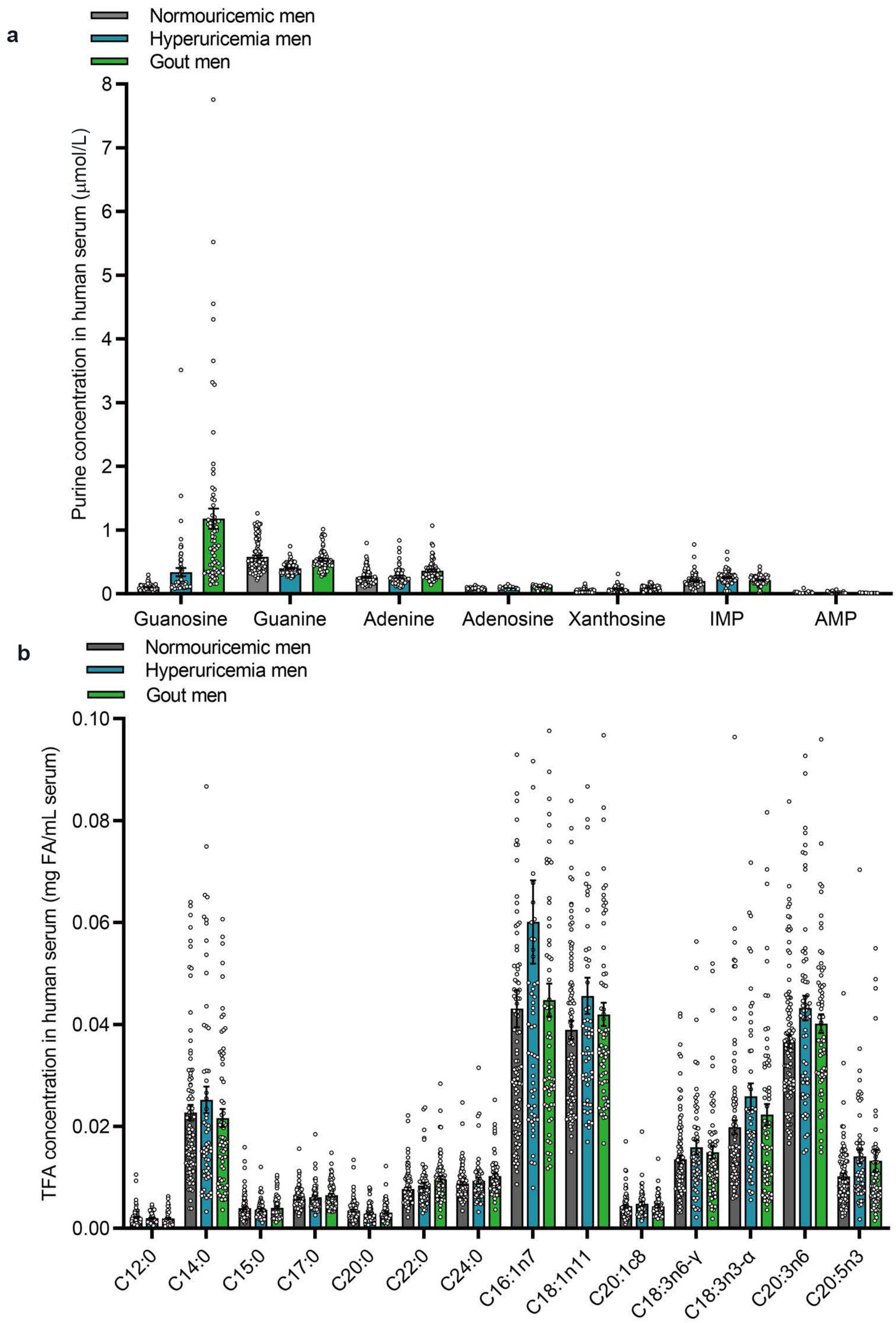

Supplementary Figure S2 Purine metabolites and fatty acids in human serum are quantified by MS. (a and b) Low content metabolites related to purine (a) and fatty acids (b) in human serum (n = 241). $^{*}P < 0.05$, $^{**}P < 0.01$, $^{***}P < 0.001$ by unpaired Mann–Whitney test (A and B).

Supplementary Figure S3 Related to Figure 2.

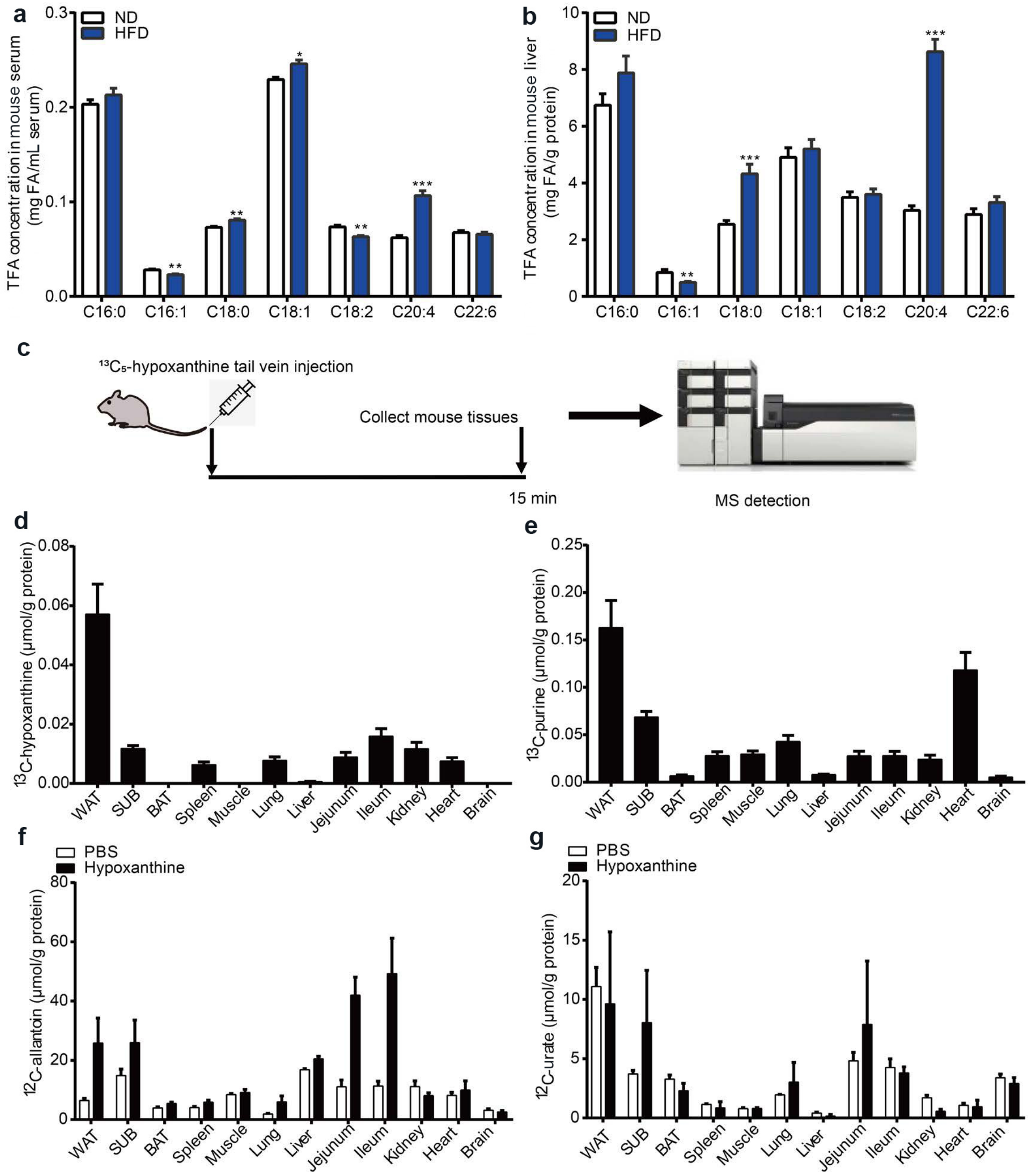

Supplementary Figure S3 Purine metabolic flow detection at mouse individual levels. (a and b) Quantitative results of fatty acids in mouse serum samples (a) and liver samples (b) (n = 10). (c) Schematic diagram of purine metabolism flow detection at mouse individual levels. For 8-week-old wild-type male mice, at a dose of 50 μmol/kg body weight, hypoxanthine solution ($^{13}C_5$-hypoxanthine:^{12}C-hypoxanthine=1:9) was injected into the tail vein of the experimental group at 0 min, and the control group was injected with the same volume of 1 × PBS. The whole-body tissues of the mice were collected after 15 min. According to the transformation mechanism of purine metabolites, the labeled and unlabeled purine metabolites were measured by LC-MS (n = 3). (d and e) Quantitative results of $^{13}C_5$-hypoxantine (d) and $^{13}C_5$-purines (e) in mouse tissues. (f and g) Quantitative results of ^{12}C-allantoin (f) and ^{12}C-urate (g) in mouse tissues (n = 3). $^{*}P < 0.05$, $^{**}P < 0.01$, $^{***}P < 0.001$ by two-tailed unpaired Student's t-test (a and b).

Supplementary Figure S4 Related to Figure 3.

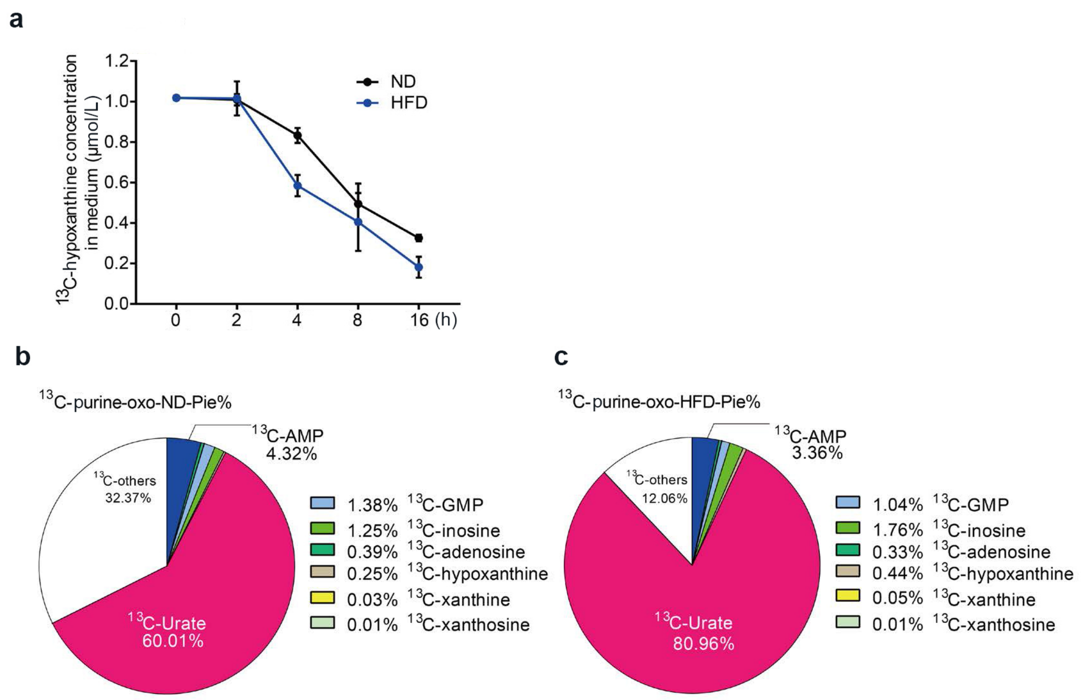

Supplementary Figure S4 Purine metabolic flow detection in mice hepatocytes. (a) The content of $^{13}C_5$-hypoxanthine in the media of mouse hepatocytes at different time points by LC-MS (n = 3). (b and c) Quantitative results of derived ^{13}C labeled purine metabolites after potassium oxonate and $^{13}C_5$-hypoxanthine treatment in control group (b) and HFD group (c) (n = 3).

Supplementary Figure S5 Related to Figure 4.

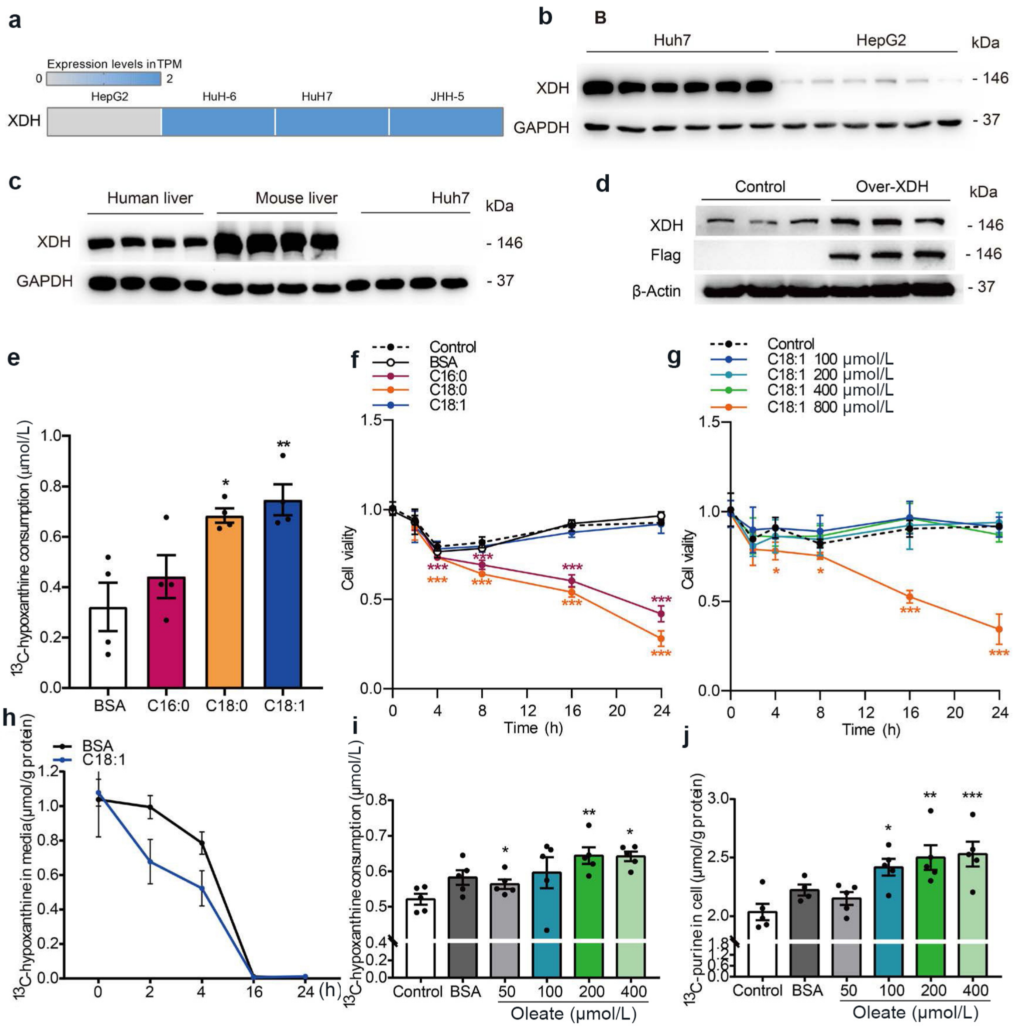

Supplementary Figure S5 Experimental design for purine metabolic flux from $^{13}C_5$-hypoxanthine in human hepatocyte lines. (a) The TPM data (1) shows higher XDH expression levels in Huh7 cells than in other hepatic cell lines. (b) The protein levels of XDH in Huh7 and HepG2 cells. (c) The protein levels of XDH in human liver, mouse liver, and Huh7 cells. (d) Validation of XDH over-expression by Western blot. (e) The $^{13}C_5$-hypoxanthine consumption is increased with palmitate, stearate, and oleate treatment (n = 4). (f) Cell proliferation capacity after stimulation of 200 μmol/L different fatty acids (n = 6). (g) Cell proliferation capacity after oleate stimulation in different doses (n = 6). (h) The content of $^{13}C_5$-hypoxanthine in the media after treatment with 200 μmol/L oleate for gradient time points. (i and j) The $^{13}C_5$-hypoxanthine consumption (i) and $^{13}C_5$-purine metabolites (j) are increased in a dose-dependent manner with oleate treatment (n = 5). $^{*}P < 0.05$, $^{**}P < 0.01$, $^{***}P < 0.001$ by two-tailed unpaired Student's t-test (e–g, i, and j).

Supplementary Figure S6 Related to Figure 4.

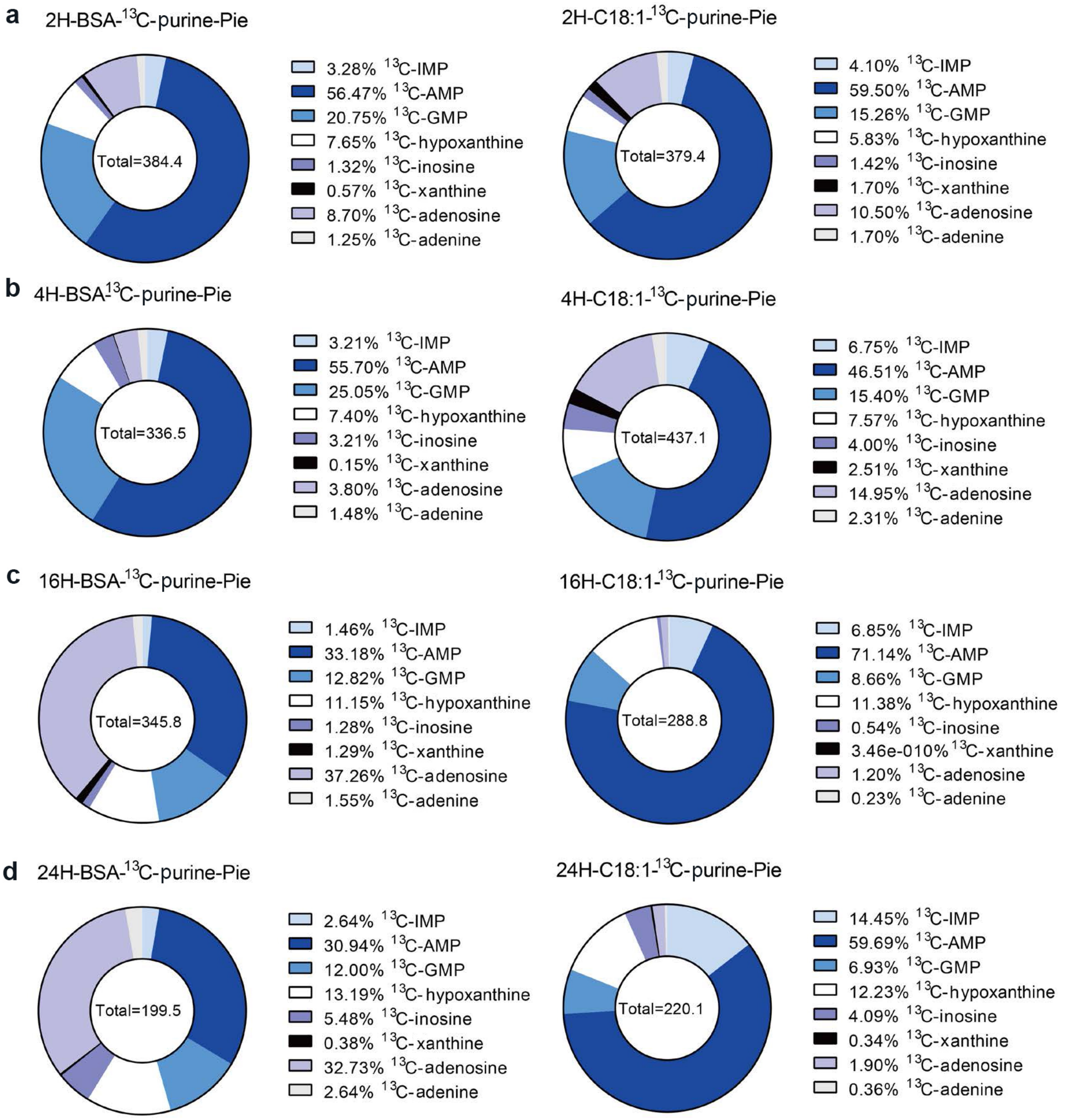

Supplementary Figure S6 The derived ^{13}C-purine metabolites of Huh7 cells in different time points with oleate incubation. (a) Huh7 cells generate more $^{13}C_5$-xanthine in oleate-treated group after incubation with $^{13}C_5$-hypoxanthine for 2 h. (b) Huh7 cells generate more $^{13}C_5$-xanthine in oleate-treated group, with an increase in total amount of the labeled products, after incubation with $^{13}C_5$-hypoxanthine for 4 h. (c) After 16 h of treatment with $^{13}C_5$-hypoxanthine, Huh7 cells produce more $^{13}C_5$-AMP and $^{13}C_5$-IMP, while the controls produce more $^{13}C_5$-adenosine. (d) Total amount of labeled products is further reduced at 24 h, with a similar trend to that observed at 16 h in both groups (n = 3).

Supplementary Figure S7 Related to Figure 5.

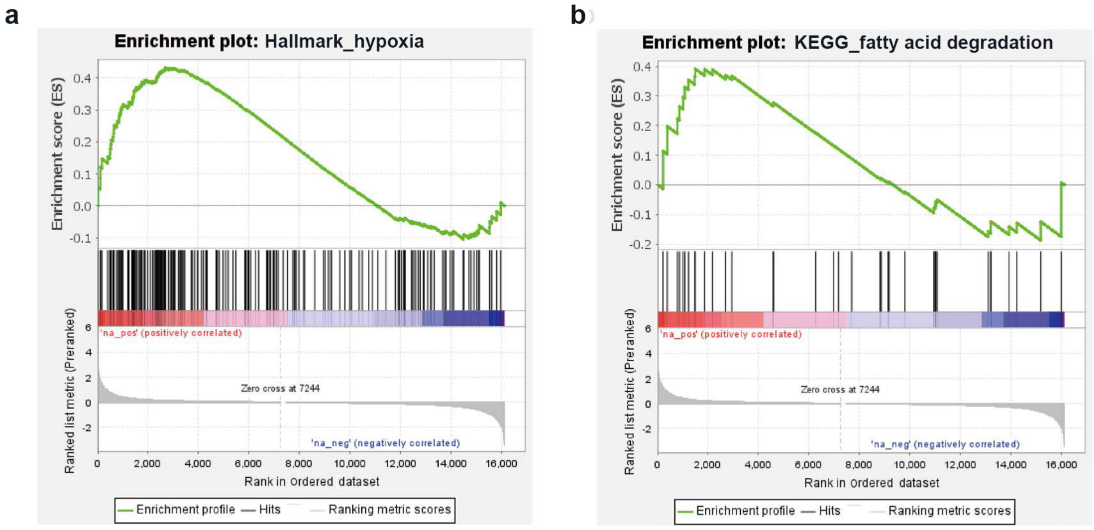

Supplementary Figure S7 GSEA enrichment plot shows enhanced hypoxia pathway and fatty acid degradation metabolism in Huh7 cells with oleate incubation. (a) GSEA enrichment plot showing significant enrichment of hypoxia signature with oleate treatment. (b) GSEA enrichment plot showing significant enrichment of the fatty acid degradation metabolism with oleate treatment (n = 3).

Supplementary Figure S8 Related to Figure 5.

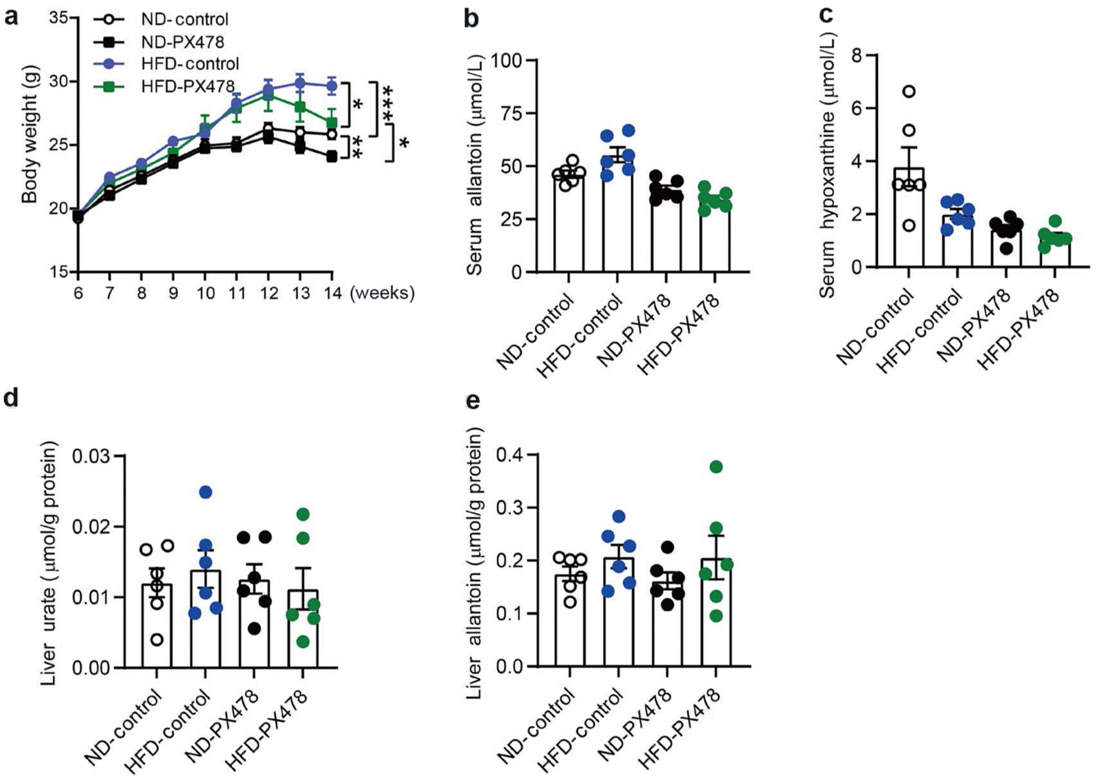

Supplementary Figure S8 The effect of PX-478 in HFD-induced HU mouse model. (a) PX-478 treatment (either in ND or HFD-fed group) could significantly decrease mouse body weight during the experimental period. (b and c) Allantoin (b) and hypoxanthine (c) in mouse serum. (d and e) Urate (d), allantoin (e) in mouse liver (n = 6). $^{*}P < 0.05$, $^{**}P < 0.01$, $^{***}P < 0.001$ by two-tailed unpaired Student's t-test (b–e).

Supplementary Figure S9 Related to Figure 6.

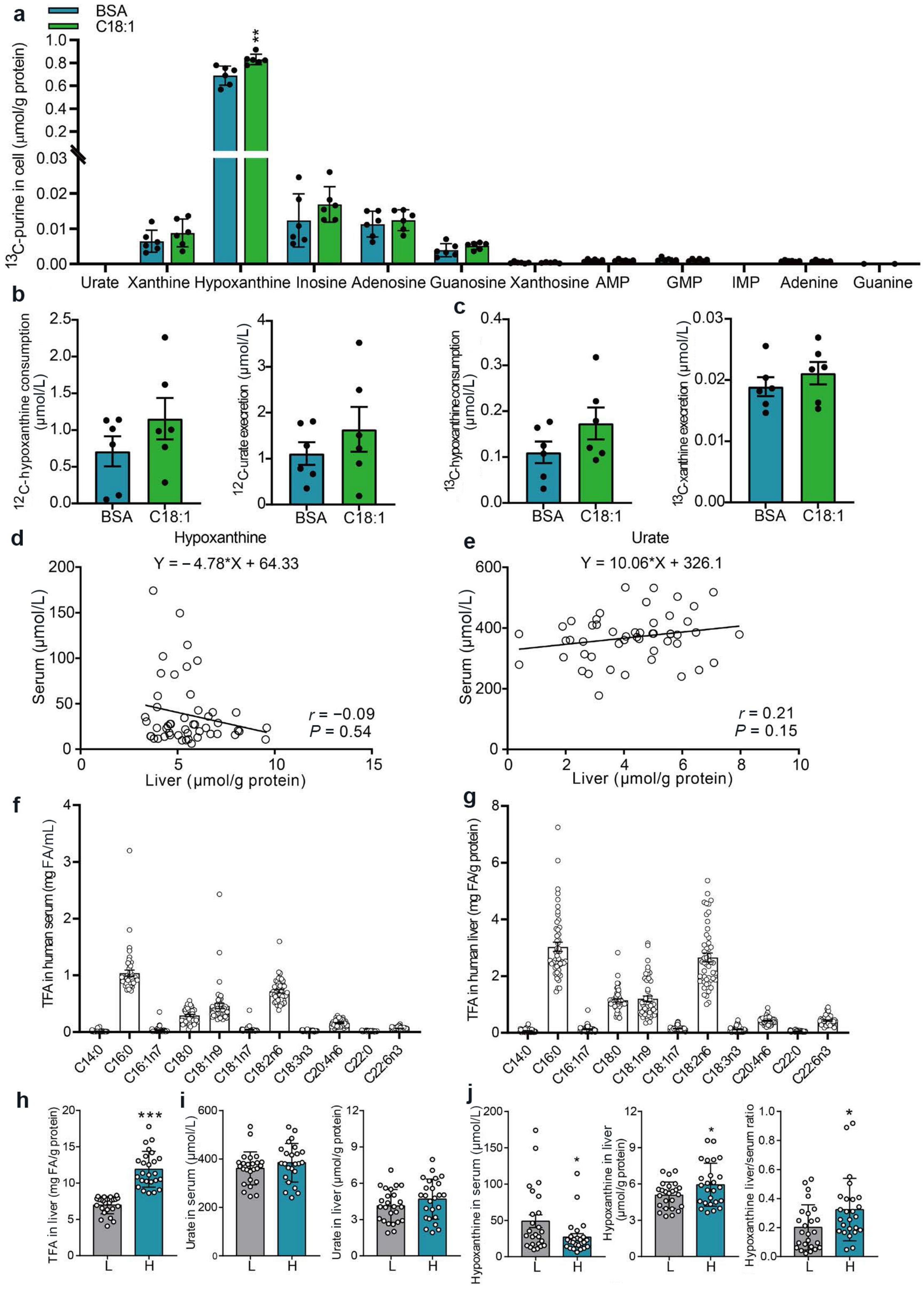

Supplementary Figure S9 Fatty acids promote human hepatocytes to consume more hypoxanthine and execrate more urate into the media. (a) Quantitative results of ^{13}C-purine metabolites in human hepatocyte. (b) Quantitative results of ^{12}C-hypoxanthine consumption and ^{12}C-urate execration in the media. (c) Quantitative results of ^{13}C-hypoxanthine consumption and ^{13}C-xanthine execration in the media (n = 6). (d and e) The correlation analysis of hypoxanthine (d) and urate (e) between

50 men's serum and liver tissues. (f and g) Quantitative total fatty acids (TFAs) in 50-paired serum (f) and liver tissues (g). (h) Fifty paired human samples are divided into 2 parts (low and high) by TFA in the liver tissues. (i) Urate levels in human serum and liver tissues. (j) Hypoxanthine levels in human serum and liver tissues and their ratio. $^{*}P < 0.05$, $^{**}P < 0.01$, $^{***}P < 0.001$ by two-tailed unpaired Student's *t*-test (a and i), and unpaired Mann–Whitney test (h and j).

Supplementary Note 1 The description of the isotope labeling assays.

We used $^{13}C_5$-hypoxanthine to trace the metabolic flux in the purine salvage pathway. Purine salvage pathway is quite different from glucose metabolism, for the number of carbon atoms in purine nucleotide didn't change. Subtraction of natural isotopes in molecules is critical for isotope labeling experiments. The general correction matrix (2) shows that the ratio of the natural +5 Da isotopologue of hypoxanthine is only 2.56675×10^{-9}, which can be ignored. Thus, the purine metabolites with +5 Da mass increment in the salvage pathway can be quantified directly by triple quadruple MS without natural stable isotope abundance correction.

We also performed a metabolic flux assay to trace the DNPB using [$^{13}C_3$,^{15}N]-serine by triple quadruple MS. There are five possible isotope labeling forms in the end nucleotides, from +1 to +5. We thus took all possible labelling form transitions into consideration, and unlabeled control samples were included to eliminate signals in ^{13}C SRM targets (3). The final MS data were corrected natural stable isotope abundance by IsoCorrectoR (4).

Supplementary Material

Materials and methods

Chemical reagents and materials

LC-MS-grade water was generated by a Milli-Q water purification system (Millipore, Bedford, MA, USA). Isopropyl alcohol (IPA), acetonitrile (ACN), methanol (MeOH), and formic acid (HCOOH) were purchased from Merck (Muskegon, MI, USA). Labeled [$^{13}C_5$]-hypoxanthine (CLM-8042) and [$^{13}C_3$,^{15}N]-serine (CNLM-474-H) were purchased from Cambridge Isotope Laboratory. Potassium oxonate (HY-17511) and fluorouracil (HY-90006) were purchased from MCE (Shanghai, China). Oleate (O7501) and palmitate (P9767) were purchased from Sigma-Aldrich. Fatty Acidree Heat Shock Bovine Serum Albumin Powder (BAH-66) was purchased from Equitech-Bio. DMEM (Gibco, C11995500 CP), penicillin-streptomycin (Gibco, 15140-122), McCoy's 5A medium (Gibco, 16600082), collagenase type I (Worthington, LS004196), collagenase type IV (Worthington, LS004188), collagen (GelTrex, Gibco), and FBS (10099-141C) were used for cell culture. Opti-MEM (Gibco, 11058-021) and Lipofectamine-3000 Transfection Kit (Invitrogen, L3000-015) were used for transfection assays.

Antibodies

Antibodies used for Western blot analysis: GAPDH (Proteintech, 60004-1-Ig, 1:5000), ENT1 (Proteintech, 11337-1-AP, 1:1000), ENT2 (Abcam, ab181192, 1:1000), NT5C2 (Abcam, ab96084, 1:1000), XDH (Abcam, ab109235, 1:1000), Flag tag (Abmart, M20008, 1:1000), β-Actin (Cell signaling, 4967S, 1:1000), HIF-1α (Cell signaling, 36169S, 1:1000), CPT1A (Abcam, ab234111, 1:1000), ACSL1 (Abcam, ab177958, 1:1000), HPRT1 (Proteintech, 15059-1-AP, 1:1000), PNP (Proteintech, 18009-1-AP, 1:1000), ADSS (Proteintech, 16373-1-AP, 1:1000), ADSL (Proteintech, 15264-1-AP, 1:1000), HRP-Goat anti mouse IgG (H+L) (Proteintech, SA00001-1, 1:5000), HRP-Goat anti rabbit IgG (H+L) (Proteintech, SA00001-2, 1:5000).

Plasmid construction

XDH expression plasmids were constructed by cloning the open reading frame of *XDH* cDNA into the multiple cloning sites of the pcDNA3.1 vector with a Flag tag.

The target sequence used in *shNT5C2* was as follows: GCCTCATACAAGGTCCTACAT. DH5α competent cells were used for transformation. The TIAN pure Midi Plasmid Kit was used to amplify and extract plasmids. All the constructs were sequenced to confirm the results.

Plasmid transfection

Huh7 cells were seeded in 6-well plates at a density of 2 × 10^5 per plate. Plasmids were transfected into cells by lipofectamine 3000 Transfection Reagent and lasted for 48 h. For isotope labeling, Huh7 cells was then incubated with [$^{13}C_5$]-hypoxanthine for 4 h. Cells were harvested for further analysis including qPCR, Western blot, or purine metabolites measurement.

Supplementary Table S1 Sequences of human primers used for qPCR analysis.

Gene name	Forward (5' to 3')	Reverse (5' to 3')
ACTIN	CATGTACGTTGCTATCCAGGC	CTCCTTAATGTCACGCACGAT
ADA	GCCTTCGACAAGCCCAAAGTA	CTCTGCTGTGTTAGCTGGGAG
ADSL1	GCTGGAGGCGATCATGGTTC	TGATAGGCAAACCCAATGTCTG
ADSSL1	ATGGGAAAGAGTACGACTTCCA	CCCGTTGCCAATGAAGGACA
APRT	GGCCGCATCGACTACATCG	CTCAGCCTTCCCGTACTCC
GDA	GCTGGAAGTAGCATAGACCTGC	TCTTCTGCAAAGTCGATGTTCTG
GMPS	ATGGCTCTGTGCAACGGAG	CCTCACTCTTCGGTCTATGACT
HPRT1	CCTGGCGTCGTGATTAGTGAT	AGACGTTCAGTCCTGTCCATAA
IMPDH2	GCGCTTACAGGCGGTATTG	AAAACATCCCGCACGCGAT
NT5C2	TCGTCGAGAAGCCTATCATCG	GGGACTCATACTCTGGGGACT
NT5C1a	GCTGTACCCTGATAGTGAGGA	CGATGAACAGGTCATAGTGGTTG
NT5C1b	GCAGCACCAAAATGCAAGAGA	CTCGGACAGAGAGTTGCGG
NT5C	ACCTGGCGGATAAAGTGGC	GGTAGGTCGTTCATCTCCCG
PNP	ATGGAGAACGGATACACCTATGA	GAGGTCGGTGCTTAGTGTGAG
PRPS1	TGGCGAAATCAACGACAGTCT	GGGACCGGCTCTTATCCTTC
XDH	TGGACCCGACGGTATCTCC	AACGGACGCCACAGACTTG
HIF-1α	ATCCATGTGACCATGAGGAAATG	TCGGCTAGTTAGGGTACACTTC
ENT1	ACTCCAAAGTCTCAGCAGCAGG	TTCCCAGACCCAGCATGAAGA
ENT2	AACATCATGGACTGGCTGGG	ATGAAGAGGGGCACGAACAG
ENT3	GTGGCCTCATTGGTGGACTT	CCTCATGTAGTACCTTCACCTGG
GLUT9	CCTCTACGGCTACAACCTGTC	AGAGTGTCTGGGTCTATTGGAC
ABCG2	CAGGTGGAGGCAAATCTTCGT	ACCCTGTTAATCCGTTCGTTTT

References

1. Barretina J, Caponigro G, Stransky N *et al*. The Cancer Cell Line Encyclopedia enables predictive modelling of anticancer drug sensitivity. *Nature* 2012;**483**:603−7.
2. Buescher JM, Antoniewicz MR, Boros LG *et al*. A roadmap for interpreting ^{13}C metabolite labeling patterns from cells. *Curr Opin Biotechnol* 2015;**34**:189−201.
3. Yuan M, Kremer DM, Huang H *et al*. *Ex vivo* and *in vivo* stable isotope labelling of central carbon metabolism and related pathways with analysis by LC-MS/MS. *Nat Protoc* 2019;**14**:313−30.
4. Heinrich P, Kohler C, Ellmann L *et al*. Correcting for natural isotope abundance and tracer impurity in MS-, MS/MS- and high-resolution-multiple-tracer-data from stable isotope labeling experiments with IsoCorrectoR. *Sci Rep* 2018;**8**:17910.
5. Pei L, Waki H, Vaitheesvaran B *et al*. NR4A orphan nuclear receptors are transcriptional regulators of hepatic glucose metabolism. *Nat Med* 2006;**12**:1048−55.
6. Li M, He X, Guo W *et al*. Aldolase B suppresses hepatocellular carcinogenesis by inhibiting G6PD and pentose phosphate pathways. *Nat Cancer* 2020;**1**:735−47.
7. Liu G, Wang N, Zhang C *et al*. Fructose-1,6-bisphosphate aldolase B depletion promotes hepatocellular carcinogenesis through activating insulin receptor signaling and lipogenesis. *Hepatology* 2021;**74**:3037−55.
8. Li R, Li Y, Kristiansen K *et al*. SOAP: short oligonucleotide alignment program. *Bioinformatics* 2008;**24**:713−4.
9. Langmead B, Salzberg SL. Fast gapped-read alignment with Bowtie 2. *Nat Met* 2012;**9**: 357−9.
10. Li B, Dewey CN. RSEM: accurate transcript quantification from RNA-Seq data with or without a reference genome. *BMC Bioinformatics.* 2011;**12:**323.
11. Subramanian A, Tamayo P, Mootha VK *et al*. Gene set enrichment analysis: a knowledge-based approach for interpreting genome-wide expression profiles. *Proc Natl Acad Sci USA* 2005;**102**:15545−50.
12. Liberzon A, Birger C, Thorvaldsdóttir H *et al*. The Molecular Signatures Database (MSigDB) hallmark gene set collection. *Cell Syst* 2015;**1**:417−25.
